# Diagnostic and immunological roles of leptin gene rs7799039 polymorphism and cytokines in COVID-19, HCV, and dual infection

**DOI:** 10.1038/s41598-026-35418-4

**Published:** 2026-02-02

**Authors:** Amany A. Sakr, Amr E. Ahmed, Nabil A. Hasona, Rasha M. Abdel-Hamid, Asmaa Salman Faisal, Elsaeed E. Shaaban, Sahar Mohamed, Abbas Mohamed Abbas, Amal A. Mohamed

**Affiliations:** 1 Department of Biotechnology and Life Sciences, Faculty of Postgraduate Studies for Advanced Sciences, Beni-Suef University, Beni-Suef, Egypt; 2 Biochemistry Department, Faculty of Science, Beni-Suef University, Beni- Suef, Egypt; 3 Clinical Pathology Department, National Cancer Institute, Cairo University, Cairo, Egypt; 4 Internal Medicine Department, Faculty of Medicine, Arish University, Arish, Egypt; 5 Internal Medicine Department, Matarya Teaching Hospital, The General Organization for Teaching Hospitals and Institute (GOTHI), Cairo, Egypt; 6 Tropical Department, National Hepatology and Tropical Medicine Research Institute (NHTMRI), The General Organization for Teaching Hospitals and Institute (GOTHI), Cairo, Egypt; 7 Biochemistry Department, Faculty of Medicine, Cairo University, Cairo, Egypt; 8 Biochemistry and Molecular Biology Department, National Hepatology and Tropical Medicine Research Institute (NHTMRI), The General Organization for Teaching Hospitals and Institute (GOTHI), Cairo, Egypt

**Keywords:** HCV, COVID-19, Cytokines, Leptin, Gene polymorphism, Immunity, Biochemistry, Biotechnology, Genetics, Molecular biology

## Abstract

The identification of reliable diagnostic biomarkers is essential for improving the early detection, prognosis, and management of viral infections. Interleukin-6 (IL-6), tumor necrosis factor-alpha (TNF-α), and Interferon-gamma (IFN-γ) are key cytokines involved in immune regulation and inflammatory responses, playing a pivotal role in the pathogenesis of hepatitis C virus (HCV), coronavirus disease 2019 (COVID-19), and co-infection with both viruses. This study aims to investigate the role of circulating leptin gene polymorphism (rs7799039, G > A), together with IL-6, TNF-α, and IFN-γ biomarkers, in the context of HCV, COVID-19, and dual HCV–COVID-19 infection. While previous research has examined rs7799039 in COVID-19, its contribution in co-infection remains unclear. By integrating cytokine profiling with genotypic analysis, this work provides additional insights into disease diagnosis, risk stratification, and the underlying immunopathological mechanisms of these infections. Biomarker levels were measured in plasma using enzyme-linked immunosorbent assay (ELISA). Nasopharyngeal swabs were collected for real-time polymerase chain reaction (RT-PCR) detection of COVID-19 ribonucleic acid (RNA), and blood samples were used for quantitative RT-PCR detection of HCV-RNA. Single nucleotide polymorphisms (SNP) genotyping assay for Leptin gene (rs7799039) was examined from genomic deoxyribonucleic acid (DNA) using RT-PCR. No cytokine correlations were found in HCV alone, but TNF-α and IFN-γ correlated in dual infections. IL-6, TNF-α, and IFN-γ levels were significantly elevated in all patient groups compared to controls, with mean ± SD values of IL-6: 199.2 ± 28 pg/mL (co-infected), 171 ± 31.9 pg/mL (COVID-19), and 46.8 ± 9 pg/mL (HCV); TNF-α: 64.6 ± 15.4 pg/mL, 33.5 ± 7.1 pg/mL, and 32.9 ± 6.2 pg/mL; IFN-γ: 166.2 ± 41.9 pg/mL, 116.8 ± 18.6 pg/mL, and 101 ± 14.4 pg/mL, respectively. TNF-α and IFN-γ were significant predictors of disease status across groups (OR = 1.107–1.482, *P* < 0.001). IL-6 was predictive only in HCV patients (OR = 1.508, *P* < 0.001). TNF-α performed well (> 85%) in all groups. IFN-γ performed well in co-infection and COVID-19 (> 83%), but was moderately accurate in HCV (about 60%). IL-6 showed perfect accuracy in co-infection and COVID-19. Genotype analysis of the leptin gene polymorphism (rs7799039, G > A) indicated that the GA genotype was the most common across all study groups. The AA genotype of the leptin gene (rs7799039, G > A) was more frequent in individuals with COVID-19, suggesting a potential association with increased disease susceptibility. Conversely, the GG genotype was more commonly observed in the control group, which may indicate a protective role against infection. Statistical analysis revealed a significant relationship between genotype and disease status (χ² = 13.52, *P* = 0.035 < 0.05). This study highlights the distinct roles of IL-6, TNF-α, and IFN-γ in HCV, COVID-19, and co-infections, with more pronounced cytokine interactions in dual infections. Furthermore, the AA genotype may be associated with increased susceptibility to COVID-19, while the GG genotype was more frequent among controls, suggesting a possible protective trend that warrants further investigation. This study suggests differential roles of IL-6, TNF-α, and IFN-γ across HCV, COVID-19, and co-infections, with indications of more pronounced cytokine interactions in dual infections. Furthermore, the GA genotype appeared more common among patients, while the GG genotype was more frequent in controls, suggesting a possible protective trend. The AA genotype showed a slight increase in COVID-19 cases, but this association was not statistically significant and should be interpreted with caution.

**Supplementary Information:**

The online version contains supplementary material available at 10.1038/s41598-026-35418-4.

## Introduction

The SARS-CoV-2 virus, responsible for the COVID-19 pandemic, poses a serious global public health threat. This emerging respiratory disease is closely associated with interferons (IFNs)^1,2^, key cytokines that initiate and modulate antiviral responses in innate immune cells, including natural killer (NK) cells^3^. Upon viral detection by pattern recognition receptors such as Toll-like receptors (TLRs), particularly TLR3 and TLR7/8, a signaling cascade leads to the production of type I IFNs (IFN-Is), which restrict viral replication and promote antiviral immunity^4,5^.

IFN-Is activate hundreds of antiviral genes, establishing a protective intracellular environment. While type I and III IFNs share overlapping gene targets^6^, type II IFNs—particularly IFN-γ—regulate a distinct set of genes^7–9^ via IFN-γ-activated sites^10^. IFN-γ, though structurally different, can influence genes also regulated by type I IFNs, suggesting shared immunological functions.

In hepatitis C virus (HCV) infection, macrophages and dendritic cells generate IFNs independently of direct infection, continuously surveilling viral components. Host interferon signaling remains central to antiviral defense, yet the mechanisms of IFN-mediated responses in HCV remain incompletely defined^11^.

COVID-19 is associated with severe respiratory damage and dysregulated immune responses^12^, including cytokine storm syndromes characterized by elevated IL-2, IL-7, IL-10, and TNF-α^1^. Lung tissues from deceased patients show increased mast cell infiltration in perivascular and septal regions^13^. Additionally, liver injury in COVID-19 has been linked to elevated IL-6 levels, hypercoagulability, and the presence of hepatic micro-thrombi^14–17^. These findings suggest a vascular basis for liver damage, although mechanisms remain unclear.

IL-6, a key cytokine in severe COVID-19, has been directly linked to hyperinflammation, organ damage, and disease severity^18–20^. It contributes to liver injury through both classical and trans-signaling via the Janus kinase signal transducer and activator of transcription (JAK/STAT) pathway. In trans-signaling, IL-6 binds to its soluble receptor (sIL-6R), activating gp130 on target cells and promoting broad immune responses, especially under inflammatory conditions^20–24^. This mechanism contributes to endothelial glycocalyx disruption, upregulation of adhesion molecules, hypercoagulability, and sinusoidal endothelial dysfunction—key features of COVID‑19–associated vascular and hepatic injury^19,25–28^.

The leptin gene plays a central role in regulating immune and metabolic pathways. A common SNP in the promoter region, rs7799039 (G > A), has been associated with altered leptin expression and immune responsiveness. Variations in this gene have been linked to chronic inflammation, liver fibrosis, metabolic dysfunction, and response to antiviral therapy in HCV, as well as disease severity in COVID-19. Despite its potential immunogenetic significance, rs7799039 has not been well-characterized in the context of viral co-infections.

Leptin acts as a pro-inflammatory cytokine and may influence viral, bacterial, and parasitic infections^29–35^. Its deficiency or resistance alters cytokine production, affecting susceptibility and inflammation^36–38^. Elevated leptin levels are associated with poorer outcomes and may explain the link between obesity and severe COVID-19^39,40^. In chronic HCV, elevated leptin is associated with hepatic steatosis^41,42^.

Previous studies have shown that the leptin gene polymorphism rs7799039 (G > A) can influence circulating leptin levels, potentially altering immune and inflammatory responses^43^. Given leptin’s role in regulating pro-inflammatory cytokines such as IL-6 and TNF-α, genetic variations at this locus may contribute to disease severity in viral infections^44^.

Accordingly, the current study aims to evaluate the potential association of LEP rs7799039 polymorphism with disease risk and its relationship with serum levels of IL-6, TNF-α, and IFN-γ. We also discuss their diagnostic utility by assessing sensitivity and specificity to identify candidate biomarkers for HCV, COVID-19, and co-infection.

The leptin promoter polymorphism rs7799039 (G > A) was selected due to its known impact on transcriptional activity and circulating leptin levels, and its prior associations with inflammatory, metabolic, and hepatic conditions. These features make it a biologically plausible candidate for evaluating immune responses in viral infections such as HCV and COVID-19.

## Patients and methods

### Study population

**Fig. 1:**
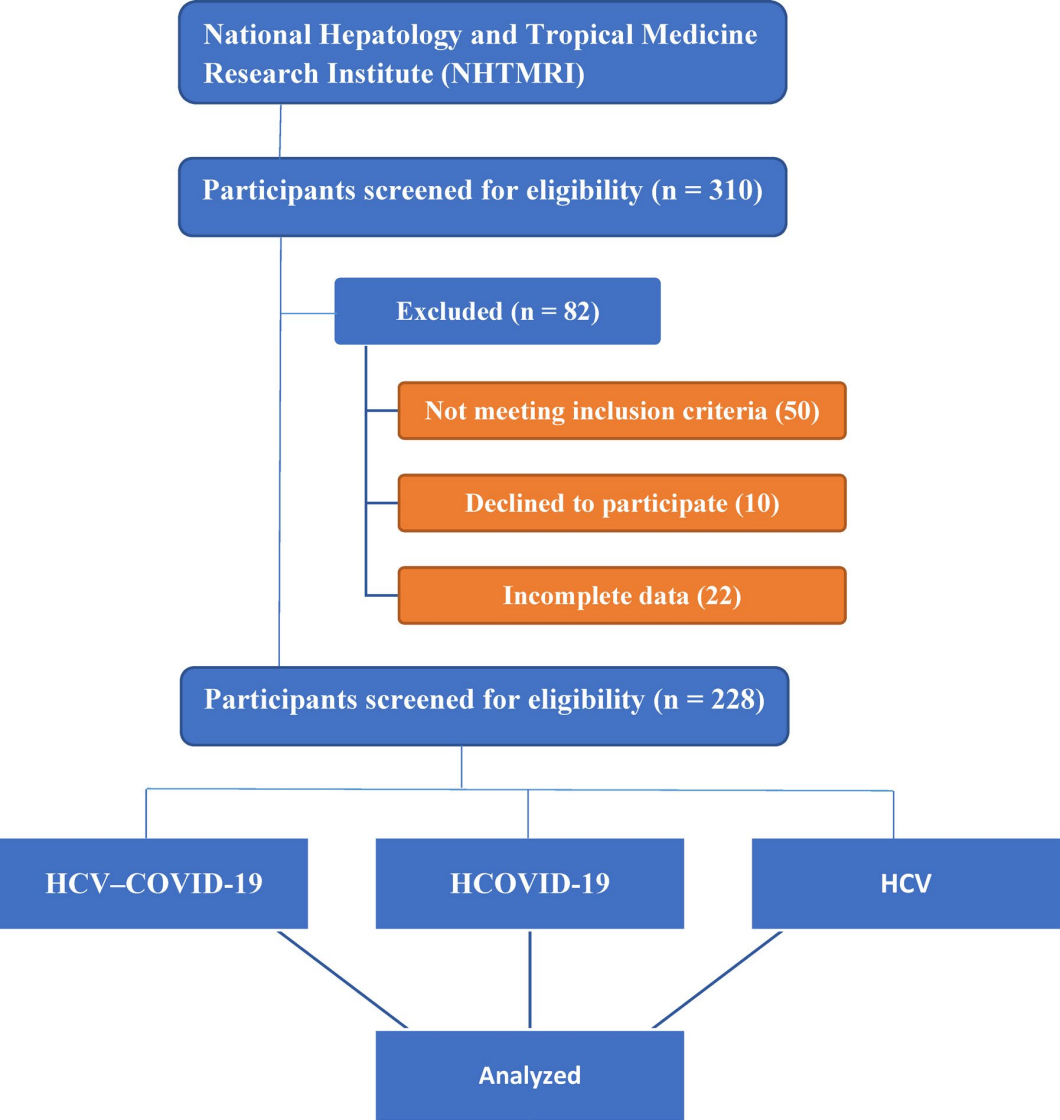
CONSORT-like flow diagram of participant recruitment and selection process. A total of 310 individuals were initially screened for eligibility at the National Hepatology and Tropical Medicine Research Institute (NHTMRI), Cairo, Egypt. Eighty-two participants were excluded due to not meeting inclusion criteria (*n* = 50), declining participation (*n* = 10), or providing incomplete data (*n* = 22). The remaining 228 eligible participants were enrolled and equally assigned into four groups (*n* = 57 each): COVID-19, HCV, co-infection, and healthy controls.

**Fig. 2:**
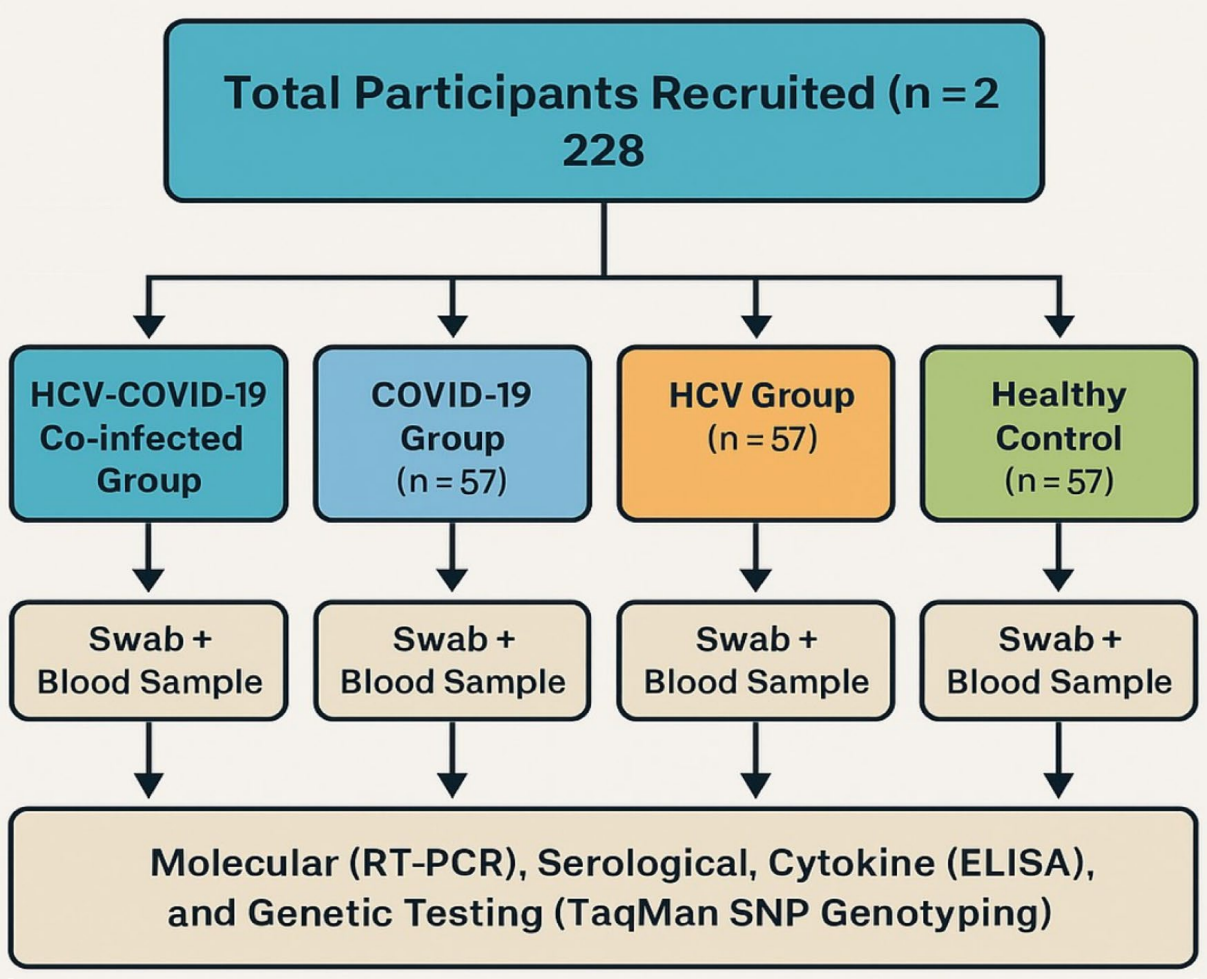
Flowchart of participant recruitment and study design. A total of 228 participants were enrolled and categorized equally into four groups: HCV-COVID-19 co-infected (*n* = 57), COVID-19 only (*n* = 57), HCV only (*n* = 57), and healthy controls (*n* = 57). Each participant provided a nasopharyngeal swab and a blood sample for molecular (RT-PCR), serological, cytokine (ELISA), and genetic (TaqMan SNP genotyping) analyses.

This prospective cross-sectional study included 228 individuals recruited from the National Hepatology and Tropical Medicine Research Institute (NHTMRI) in Cairo, Egypt. Participants provided written informed consent after receiving details about the study’s objectives, procedures, and potential risks. A comprehensive medical history, physical examination, and demographic data were collected. The study population was categorized into four groups: HCV-COVID-19 co-infected group (*n* = 57), COVID-19 group (*n* = 57), HCV group (*n* = 57), and healthy controls (*n* = 57) as shown in Figs. 1 and 2.

Sample collection and processing: Peripheral blood samples were collected using EDTA tubes for molecular and immunological assays. Plasma was separated by centrifugation at 1500 g for 10 min at 4 °C within one hour of collection. Aliquots of plasma were stored at − 80 °C until cytokine analysis.

Nasopharyngeal swabs were placed in viral transport medium and processed within 4 h for RT-PCR detection of COVID-19. All RNA and DNA extractions were performed immediately or stored at − 20 °C until analysis, in accordance with standard biosafety and biobanking protocols.

Control participants were carefully selected to ensure comparability with the patient groups. They were matched to the cases by age range and sex distribution to minimize demographic confounding. All controls tested negative for both HCV (by serology) and COVID-19 (by RT-PCR) and had no history of chronic liver disease, inflammatory disorders, or other immunological conditions. This selection strategy was implemented to establish a reliable baseline for evaluating cytokine and genetic marker differences across disease groups. All participants were of Egyptian ethnicity, reflecting the population typically served by the NHTMRI, a national referral center receiving patients from across the country.

### Materials and methods

#### Sample collection and eligibility criteria

Each participant provided two biological samples: (1) a nasopharyngeal swab for detection of severe acute COVID-19 RNA using RT-PCR, and (2) a 5 mL peripheral venous blood sample collected after overnight fasting for serological and biochemical analysis.

Eligible participants were aged ≥ 21 years and had detectable HCV-RNA levels > 12 IU/mL for at least six months without prior antiviral therapy for hepatocellular carcinoma. The control group consisted of healthy individuals negative for both HCV (by serology) and COVID-19 (by RT-PCR), with no history of chronic liver disease, inflammatory or autoimmune disorders.

Participants with any of the following conditions were excluded: human immunodeficiency virus (HIV) infection, cardiovascular disease, thyroid dysfunction, diabetes mellitus, alcohol consumption, active schistosomiasis, hepatitis B virus (HBV) infection, or history of interferon therapy.

To minimize confounding effects, individuals with diabetes and cardiovascular disease were excluded due to their known associations with chronic inflammation and altered cytokine profiles. While this exclusion may affect generalizability, it ensures more specific evaluation of infection-related immunological changes.

#### Hematological and biochemical analyses

Clinical and laboratory data were obtained from medical records. Complete blood count (CBC) was performed on whole blood samples collected in K₂EDTA anticoagulant tubes using the Swelab Alfa Basic Cell Counter (Boule Medical AB, Sweden). Liver and kidney function tests were performed using the Beckman CX4 Chemical Analyzer (Beckman Coulter Inc., USA), which operates on the principle of spectrophotometric detection, where enzymatic or colorimetric reactions are measured by absorbance at specific wavelengths.

#### HCV-RNA detection

HCV-RNA quantification was performed using a two-step protocol. Viral RNA was first extracted using the QIAamp^®^ Viral RNA Mini Kit (Qiagen, Germany), followed by amplification using the HCV RG RT-PCR Kit on the Rotor-Gene Q 5plex System (Qiagen, Germany), according to the manufacturer’s instructions.

#### COVID-19 RNA detection

Nasopharyngeal swabs were analyzed using RT-PCR for detection of COVID-19 RNA targeting the ORF1ab and N genes. A validated COVID-19 detection kit (manufacturer and catalog number provided in figure legend) was used in accordance with the manufacturer’s instructions. In cases of initial negative results, a second confirmatory test was performed within 24–48 h.

#### Cytokine quantification (IL-6, TNF-α, and IFN-γ)

Plasma samples were obtained by centrifuging whole blood at 1500×g for 10 min at 4 °C and stored at − 80 °C until analysis. Concentrations of IL-6, TNF-α, and IFN-γ were measured using commercially available enzyme-linked immunosorbent assay (ELISA) kits (Bioassay Technology Laboratory, China). All assays were conducted in duplicate according to the manufacturer’s protocols to ensure accuracy and reproducibility.

Serum levels IL-6, TNF-α, IFN-γ, and leptin were measured using validated sandwich ELISA kits. IL-6 was quantified using the Quantikine^®^ ELISA kit from R&D Systems (Minneapolis, MN, USA; Catalog No. D6050), which has a detection range of 3.13–300 pg/mL, a sensitivity of < 0.7 pg/mL, intra-assay coefficients of variation (CVs) between 2 and 4.2%, and inter-assay CVs between 3.3 and 6.4%. TNF-α was measured using the Quantikine^®^ ELISA kit from R&D Systems (Catalog No. DTA00D), with a detection range of 15.6–1000 pg/mL, a sensitivity of 2.09–6.23 pg/mL, intra-assay CVs of 2.2–3%, and inter-assay CVs of 7.3–8.4%. IFN-γ levels were determined using the R&D Systems Quantikine^®^ ELISA kit (Catalog No. DIF50), which provides a detection range of 15.6–1000 pg/mL, a sensitivity of < 8 pg/mL, intra-assay CVs of 2.6–4.7%, and inter-assay CVs of 3.7–7.8%. Leptin concentrations were measured using an ELISA kit from Bioassay Technology Laboratory (DRG International, Inc., USA; Catalog No EIA-2395), with a detection range of 0–100 ng/mL, a sensitivity of 1 ng/mL, and intra-assay CVs of 5.95–6.91% and inter-assay CVs of 8.66–11.55%.

Serum concentrations of IL-6, TNF-α, and IFN-γ were quantified using commercially available Quantikine^®^ ELISA kits (R&D Systems, Minneapolis, MN, USA) according to the manufacturer’s protocols. The analytical performance of the assays was as follows: for IL-6, the limit of detection (LOD) was < 0.70 pg/mL with intra- and inter-assay coefficients of variation (CVs) ranging from 1.7% to 6.4%; for TNF-α, the LOD was 4.00 pg/mL (range 2.09–6.23 pg/mL) with intra- and inter-assay CVs between 2.2% and 8.4%; and for IFN-γ, the LOD was approximately 8 pg/mL, with intra- and inter-assay CVs below 10%. Table T1 (in the supplementary files) providing full details of all laboratory kits, reagents, and analytical performance characteristics.

### DNA extraction and genotyping test

Genomic deoxyribonucleic acid (DNA) was extracted from whole blood samples using the QIAamp DNA Blood Mini Kit (QIAGEN, Hilden, Germany) as per the manufacturer’s instructions. DNA concentration and purity were evaluated using the NanoDrop^®^ 2000 spectrophotometer (Thermo Fisher Scientific, USA).

The leptin gene polymorphism rs7799039 (G > A), located on chromosome 7:128128730 (GRCh38), was genotyped using a predesigned TaqMan^®^ SNP Genotyping Assay (Assay ID: C___8722581_10; Thermo Fisher Scientific, USA). This single nucleotide polymorphism is well-documented in the NCBI dbSNP and ClinVar databases and has been associated with altered leptin expression and increased susceptibility to obesity, insulin resistance, and inflammatory conditions. The risk allele is A, with a global minor allele frequency (MAF) of approximately 0.39, though regional variation exists (e.g., EUR: 0.47; SAS: 0.44; AFR: 0.28). The genotyping assay uses allele-specific fluorescent probes; however, the primer and probe sequences are proprietary and not publicly disclosed by the manufacturer. Since the analysis was performed on genomic DNA, a reverse transcription (RT) primer was not required. All SNP-related information was retrieved from the NCBI database^45^. All reactions were carried out on the Applied Biosystems^®^ StepOne™ Real-Time PCR System. The cycling conditions were as follows: an initial denaturation step at 95 °C for 10 min, followed by 40 cycles of 95 °C for 15 s and 60 °C for 1 min.

TaqMan^®^ SNP Genotyping Assay (Thermo Fisher) was used. Each run included positive and negative controls. 10% of samples were randomly re-genotyped; concordance was 100%. Genotyping success rate was > 98%.

## Statistical analysis

Statistical analysis was performed to evaluate data distribution and explore relationships among variables. Normality was assessed using the Shapiro-Wilk test. Normally distributed variables were analyzed using Student’s t-test. Non-normally distributed variables were analyzed using Mann-Whitney U, where the samples are independent in our study. These tests were applied to cytokine levels and other clinical parameters when parametric assumptions were violated. To control for the false discovery rate due to multiple comparisons, P-values were adjusted using the Benjamini-Hochberg Discovery Rate (FDR) method. This correction was applied to correlation analyses and groupwise cytokine comparisons.

Pearson’s correlation coefficient was employed to determine associations between continuous variables. The diagnostic utility of blood biochemical markers was assessed using Receiver Operating Characteristic (ROC) curve analysis, with the area under the curve (AUC) calculated at a 95% CI. The Youden Index was utilized to identify optimal cutoff values, with sensitivity, specificity, and likelihood ratios subsequently calculated. Univariate and multivariate logistic regression models were applied to determine the predictive value of IL-6, TNF-α, and IFN-γ levels for identifying the risk and likelihood of HCV infection, COVID-19, and HCV-COVID-19 co-infection. Regression results were reported as B coefficients with 95% CI to quantify their effect. Statistical significance was set at a two-tailed P-value of < 0.05. All analyses and data visualizations were performed using R programming, employing the pheatmap and ggplot2 packages to ensure clarity and reproducibility in biomarker-based diagnostics.

To address potential small-sample bias and separation problems in genotype and cytokine comparisons, Firth’s penalized logistic regression was applied using the logistf package (see Heinze & Schemper, 2002^46^. The association between IL-6 levels and infection status (HCV, COVID-19, and co-infection vs. controls) was re-evaluated using penalized maximum likelihood estimates, and profile-likelihood confidence intervals were reported.

To validate the diagnostic performance of IL-6, ROC curve analysis was repeated using 2000-iteration bootstrap resampling to obtain bias-corrected 95% confidence intervals for the AUC. Optimal diagnostic thresholds were determined using Youden’s index, and diagnostic accuracy metrics were recalculated, including sensitivity, specificity, positive predictive value (PPV), and negative predictive value (NPV).

To further assess model robustness, 10-fold cross-validation was performed using a binomial logistic regression classifier including IL-6, age, sex, and BMI as predictors. Cross-validated AUC values were reported to evaluate the stability and generalizability of the model.

All multivariable logistic regression models were adjusted for age, sex, and BMI to account for adiposity-related confounding. BMI was included as a continuous variable. Sensitivity analyses stratified by BMI tertiles were also performed to evaluate the robustness of the associations.

## Results

### Evaluation of blood, liver, and kidney test outcomes in patients

Tables 1 and 2 show no significant differences in gender, age, or ESR (at 1 h and 2 h) across all groups, indicating these variables are not confounding factors. The chi-square test was applied to assess differences in gender distribution between each patient group and the control group. The results showed no statistically significant differences in gender proportions across any comparison: HCV-COVID-19 vs. control (χ² = 0.328, *P* = 0.567), COVID-19 vs. control (χ² = 0.147, *P* = 0.702), and HCV vs. control (χ² = 0.037, *P* = 0.847). Although BMI values were statistically lower in the patient groups compared to the control group, the actual differences were modest. Mean BMI scores ranged from 30.68 to 32.98 across all groups, with no group falling below the overweight threshold. This indicates that all participants, including infected and control groups, were classified as overweight or obese, and the variations in BMI are unlikely to be clinically significant. The HCV group showed some increase in Hb level (Table 2). Among the laboratory parameters evaluated, a statistically significant elevation in INR (Table 3) was observed only in the HCV group compared to the controls (mean ± SD: 1.31 ± 0.19; *P* = 0.001 < 0.05), suggesting impaired hepatic synthetic function, which is commonly associated with advanced liver disease and may indirectly reflect fibrosis. INR levels in the COVID-19 and HCV–COVID-19 co-infected groups, however, did not show statistically significant changes. Although direct fibrosis staging was not available in this study, INR is frequently used as a surrogate marker of liver dysfunction in chronic HCV infection. Since LDH (Table 3) is a marker of tissue damage and inflammation, its lower levels in the HCV group (168.65 ± 18.8 U/L; *P* = 0.006) may reflect the chronic, non-acute nature of the infection, which is typically associated with diminished immune-inflammatory activation. This suggests a relatively muted inflammatory response and limited hepatocellular injury in the absence of acute exacerbation or superinfection.

**Table 1:** Demographic characteristics of the study population across HCV-COVID-19 co-infected, COVID-19, HCV, and control groups. Data are presented as number and percentage for categorical variables and mean ± standard deviation for continuous variables. P-values reflect comparisons with the control group.

Parameters	HCV-COVID-19 group (*n* = 57)		COVID-19 group (*n* = 57)		HCV group (*n* = 57)		Control group (*n* = 57)
	Mean ± SD	*P*-value	Mean ± SD	*P*-value	Mean ± SD	*P*-value	Mean ± SD
Gender Male, n (%) Female, n (%)	32 (56%) 25 (44%)	0.450	33 (58%) 24 (42%)	0.569	34 (60%) 23 (40%)	0.567	36 (63%) 21 (37%)
Age (year)	50 ± 11.44	0.835	46.67 ± 10.7	0.137	48.12 ± 12.18	0.702	49.58 ± 10.04
BMI (kg/m^2^)	30.68 ± 5.12	0.011	31.28 ± 4.79	0.05	31.53 ± 5.17	0.847	32.9825 ± 4.36

BMI, body mass index; SD, standard deviation.

**Table 2:** Hematological and inflammatory parameters among patient groups and controls. P-values indicate statistical comparisons versus the control group.

Parameters	HCV-COVID-19 group (*n* = 57)		COVID-19 group (*n* = 57)		HCV group (*n* = 57)		Control group (*n* = 57)
	Mean ± SD	*P*-value	Mean ± SD	*P*-value	Mean ± SD	*P*-value	Mean ± SD
Hb (g/dl)	12.06 ± 4.54	0.333	11.65 ± 1.2	0.426	12.39 ± 1.31	0.001	11.4316 ± 1.69
ESR/1hour (mm)	14.32 ± 9.42	0.133	18.4 ± 10	0.529	13.91 ± 7.47	0.062	17.1754 ± 10.74
ESR/2hours (mm)	32 ± 20.98	0.105	39.39 ± 18.1	0.906	32.02 ± 18.02	0.086	38.9123 ± 24.11
O_2_-Saturation (%)	93.61 ± 4.45	< 0.001	94.81 ± 3.44	< 0.001	97.95 ± 1.06	0.26	98.1579 ± 0.92

Hb, hemoglobin; ESR, erythrocyte sedimentation rate; O₂, oxygen saturation; SD, standard deviation.

**Table 3:** Demographic and laboratory findings liver function and tissue injury markers across study groups. P-values indicate statistical comparisons versus the control group.

Parameters	HCV-COVID-19 group (*n* = 57)		COVID-19 group (*n* = 57)		HCV group (*n* = 57)		Control group (*n* = 57)
	Mean ± SD	*P*-value	Mean ± SD	*P*-value	Mean ± SD	*P*-value	Mean ± SD
INR	1.19 ± 0.08	0.759	1.17 ± 0.11	0.849	1.31 ± 0.19	0.001	1.1767 ± 0.23
LDH (U/L)	176.44 ± 35.1	0.299	183.16 ± 29	0.984	168.65 ± 18.8	0.006	183.28 ± 34.92

INR, international normalized ratio; LDH, lactate dehydrogenase; SD, standard deviation.

Oxygen saturation (Table 2) was significantly lower in both the COVID-19 and co-infected group (*P* < 0.001) but not in the HCV group. ESR values showed no significant differences among groups. Table 4 presents cytokine levels and their statistical comparisons. IL-6, TNF-α, and IFN-γ levels were significantly elevated in all patient groups compared to controls (*P* < 0.001), with the highest levels observed in co-infected individuals.

**Fig. 3:**
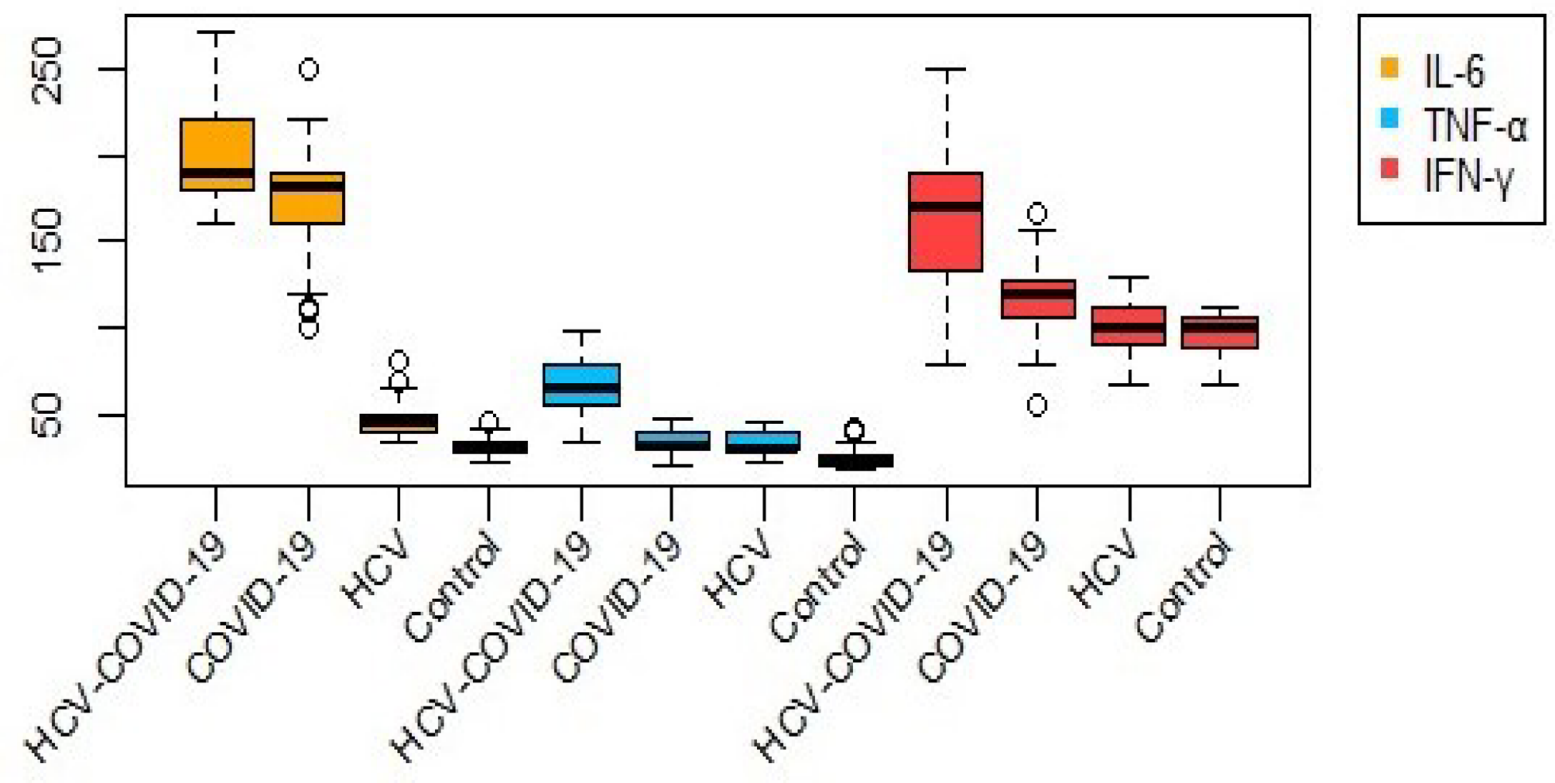
Box-plots showing serum concentrations of interleukin-6 (IL-6), tumor necrosis factor-α (TNF-α), and interferon-γ (IFN-γ) in HCV-COVID-19, COVID-19, HCV, and control groups. Each box represents the interquartile range (IQR), with the horizontal line indicating the median, and whiskers denoting data dispersion. Outliers are displayed as individual points. Distinct color coding (orange for IL-6, blue for TNF-α, and red for IFN-γ) highlights cytokine variability and overlap between the studied groups. .

As shown in Figure 3, IL-6 levels were markedly elevated in the HCV-COVID-19 group compared with COVID-19, HCV, and control groups, reflecting a strong inflammatory response associated with co-infection. TNF-α concentrations showed moderate variation among groups, with slightly higher values in HCV-COVID-19 patients. IFN-γ levels were also significantly higher in the HCV-COVID-19 group, suggesting enhanced immune activation. Overall, the box-plots demonstrate clear differences in cytokine profiles across groups, with partial overlap indicating inter-individual variability in immune responses.

**Fig. 4:**
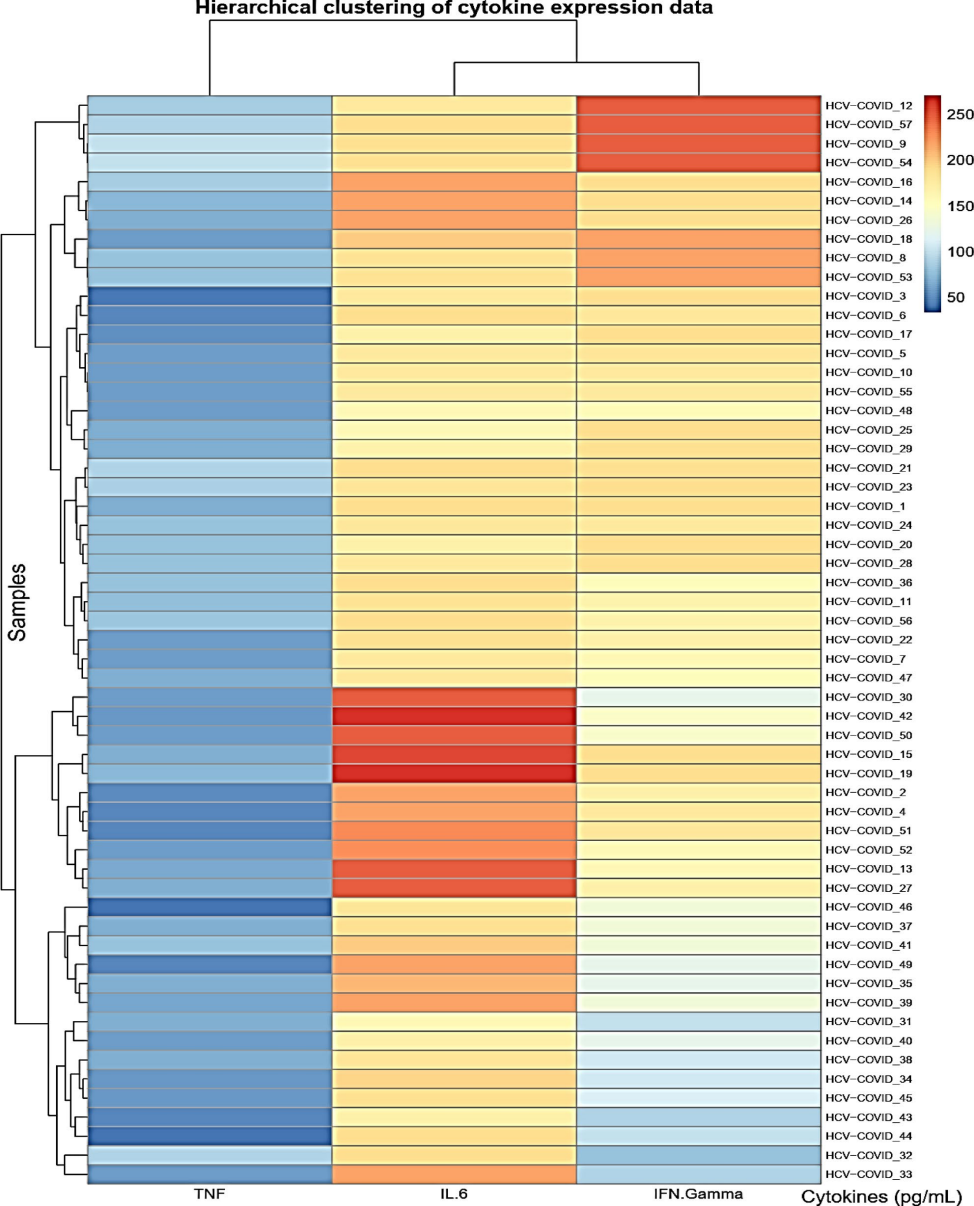
Heatmap showing the expression profiles of pro-inflammatory cytokines (TNF-α, IL-6, and IFN-γ) in dual HCV-COVID-19 group. Each row represents an individual patient sample, and each column represents a cytokine. Color intensity reflects relative expression levels, with red indicating higher expression and blue indicating lower expression. Hierarchical clustering was applied to both samples and cytokines to highlight patterns and similarities in cytokine expression across the cohort.

**Fig. 5:**
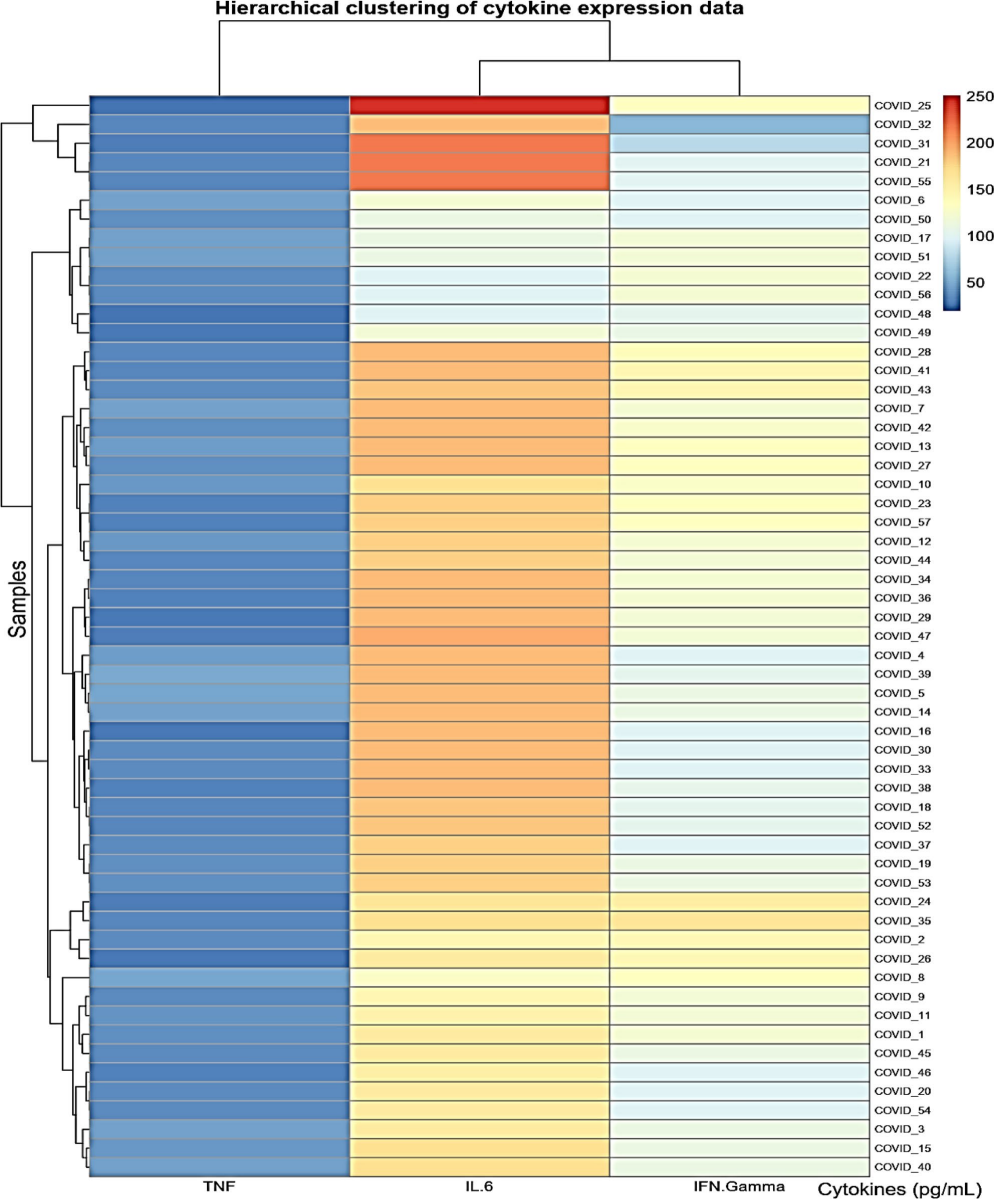
Heatmap showing the expression profiles of pro-inflammatory cytokines (TNF-α, IL-6, and IFN-γ) in COVID-19 group. Each row represents an individual patient sample, and each column represents a cytokine. Color intensity reflects relative expression levels, with red indicating higher expression and blue indicating lower expression. Hierarchical clustering was applied to both samples and cytokines to highlight patterns and similarities in cytokine expression across the cohort.

**Fig. 6:**
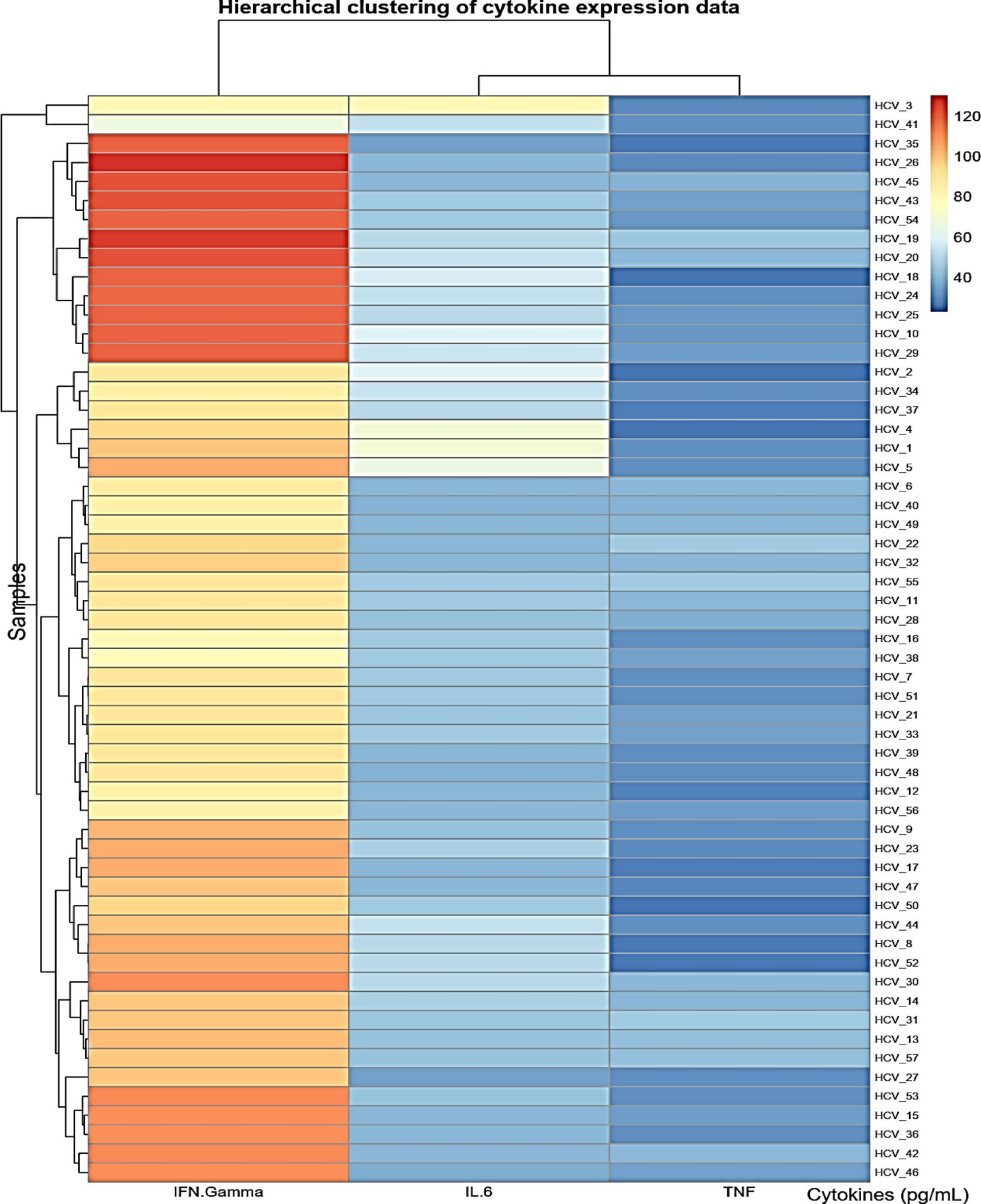
Heatmap showing the expression profiles of pro-inflammatory cytokines (TNF-α, IL-6, and IFN-γ) in HCV group. Each row represents an individual patient sample, and each column represents a cytokine. Color intensity reflects relative expression levels, with red indicating higher expression and blue indicating lower expression. Hierarchical clustering was applied to both samples and cytokines to highlight patterns and similarities in cytokine expression across the cohort.

**Fig. 7:**
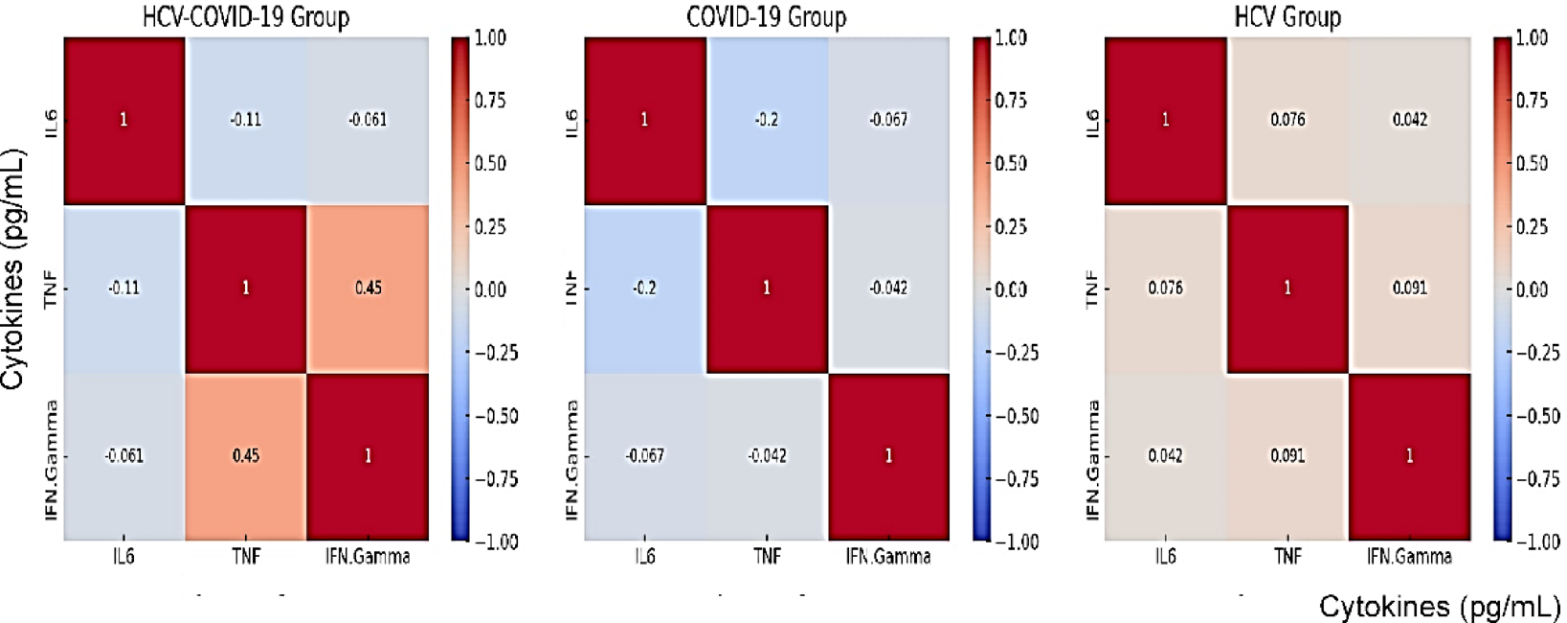
Heatmap representation of Pearson correlation coefficients among IL-6, TNF-α, and IFN-γ cytokines in three study groups: HCV-COVID-19 co-infected patients, COVID-19-only patients, and HCV-only patients. Cytokine concentrations are measured in pecograms per millilliter (pg/mL). A strong positive correlation between TNF-α and IFN-γ was observed only in the co-infected group (*r* = 0.450, *p* < 0.01), suggesting a synergistic inflammatory interaction specific to dual infection. Other correlations across groups were weak and statistically non-significant.

**Fig. 8:**
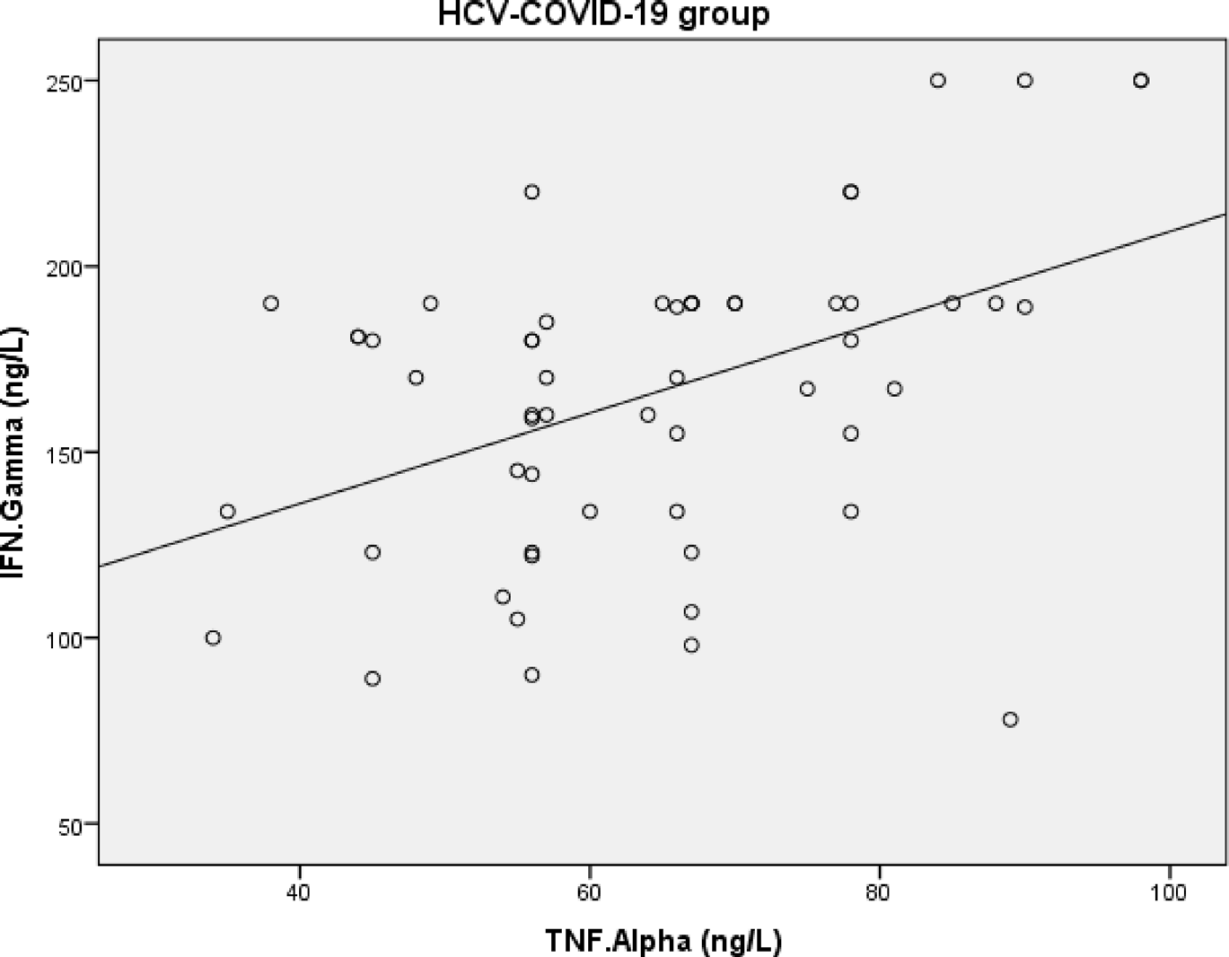
Scatter plot showing the relationship between TNF-α and IFN-γ levels in the HCV-COVID-19 co-infected group. Cytokine concentrations are measured in nanograms per liter (pg/mL). A moderate and statistically significant positive correlation was observed (*r* = 0.45, *P* < 0.01), indicating that higher levels of TNF-α are associated with increased IFN-γ expression in co-infected individuals. This relationship suggests a possible synergistic pro-inflammatory response unique to dual infection.

Heatmap visualizations for biomarkers in cases is conducted in Figs. 4, 5, and 6 utilizing the R-programming packages. The HCV-COVID-19 group (Fig. 4) shows the highest levels of IL-6, TNF-α, and IFN-γ. This suggests that co-infected patients experience greater immune activation and inflammation, likely due to the combined effects of both viral infections. COVID-19 patients (Fig. 5) show a higher immune response than HCV alone: the COVID-19 group has higher levels than the HCV group for all three biomarkers. This aligns with the known pro-inflammatory response in COVID-19, where IL-6 and TNF-α play a key role in cytokine storms. HCV alone (Fig. 6) shows the lowest inflammatory markers: the HCV group has the lowest IL-6, TNF-α, and IFN-γ levels. This suggests that HCV alone induces a milder inflammatory response compared to COVID-19 or co-infection. The heatmap in Fig. 7 presents Pearson correlation coefficients among three inflammatory markers: IL-6, TNF-α, and IFN-γ. No significant correlations were found among IL-6, TNF-α, and IFN-γ in HCV patients. Figure 7 may show a moderate correlation between TNF-α and IFN-γ in the case of co-infection indicating both inflammatory and antiviral responses. Weaker correlations in the HCV group suggest a different immune activation pattern compared to COVID-19 and co-infected patients. Chronic HCV infection doesn’t induce a strong cytokine storm, making IL-6 and TNF-α less correlated. Unlike in dual HCV-COVID-19 patients (where TNF-α and IFN-γ showed a moderate positive correlation with r = 0.45, P < 0.001), in HCV alone, the cytokine relationships appear weak and independent (Fig. 8). This suggests that inflammation and immune response mechanisms in HCV alone are different from those in HCV-COVID-19 co-infection. In the HCV group, Pearson correlation analysis showed no significant associations among IL-6, TNF-α, and IFN-γ levels. The correlation between IL-6 and TNF-α was weak (r = 0.076, P = 0.543), as was the correlation between IL-6 and IFN-γ (r = 0.042, P = 0.736). Similarly, the correlation between TNF-α and IFN-γ was also minimal and statistically non-significant (r = 0.091, P = 0.463). These findings confirm that there were no meaningful linear relationships among the cytokines in HCV patients.

**Table 4:** The effect of inflammatory factors IL-6, TNF-α, and interferon-Gamma (IFN-γ) on patients infected with COVID-19 only, HCV only, or both viruses. IL-6: in patients with COVID-19 only, IL-6 levels are very high and are associated with cytokine storm and disease severity. in patients with HCV only, IL-6 levels are also elevated but lower compared to COVID-19, and are linked to chronic liver inflammation. in patients infected with both viruses, IL-6 levels are significantly increased due to the combination of acute and chronic inflammation. TNF-α: in COVID-19 only patients, TNF-α is markedly elevated and contributes to the acute inflammatory response. in HCV patients, TNF-α remains elevated and persistent because of chronic inflammation. in co-infected patients, TNF-α increases further with more severe inflammatory response. IFN-γ: at the onset of COVID-19 infection, interferon levels rise to stimulate the antiviral immune response. in HCV cases, interferon levels May be low or impaired due to the virus’s ability to evade the immune system. in co-infections, there is a mixed effect with dysregulation of immune response.

Markers	HCV-COVID-19 group		COVID group		HCV group		Control group
	Mean ± SD	*P*-value	Mean ± SD	*P*-value	Mean ± SD	*P*-value	Mean ± SD
IL-6 (pg/mL)	199.21 ± 28.04	< 0.001	171.04 ± 31.94	< 0.001	46.77 ± 9.03	< 0.001	31.84 ± 5.5
TNF-α (pg/mL)	64.63 ± 15.45	< 0.001	33.47 ± 7.09	< 0.001	32.89 ± 6.15	< 0.001	24.47 ± 5.33
IFN-γ (pg/mL)	166.18 ± 41.86	< 0.001	116.81 ± 18.61	< 0.001	101 ± 14.41	0.027	95.23 ± 12.92

Interleukin-6 (IL-6), tumor necrosis factor-alpha (TNF-α), and Interferon-gamma (IFN-γ); SD, standard deviation.

**Table 5:** Logistic regression analysis of cytokine levels (IL-6, TNF-$\:\alpha\:$, and IFN-$\:\gamma\:$) as predictors for distinguishing HCV-COVID-19 co-infection, COVID-19, and HCV infection. The table presents regression coefficients (B), standard error (Std. Error), P-values, odds ratios (OR), and 95% confidence intervals (CI) for each cytokine in the respective disease groups. Significant predictors (*P* < 0.05) are highlighted, and extremely wide confidence intervals indicate model instability for IL-6 in some groups.

Group	Variables	B	Std. Error	P-value	OR	95% CI for OR	
						Lower Bound	Upper Bound
HCV-COVID-19	IL-6 (pg/mL)	0.312	47.458	0.995	1.366	0	3.404E + 40
	TNF-$\:\alpha\:$ (pg/mL)	0.393	0.116	0.001	1.482	1.18	1.86
	IFN-$\:\gamma\:$ (pg/mL)	0.101	0.024	< 0.001	1.107	1.056	1.16
COVID-19	IL-6 (pg/mL)	0.606	63.442	0.992	1.833	0	1.843E + 54
	TNF-$\:\alpha\:$ (pg/mL)	0.232	0.054	< 0.001	1.261	1.156	1.377
	IFN-$\:\gamma\:$ (pg/mL)	0.102	0.021	< 0.001	1.107	1.062	1.154
HCV	IL-6 (pg/mL)	0.411	0.078	< 0.001	1.508	1.293	1.759
	TNF-$\:\alpha\:$ (pg/mL)	0.254	0.049	< 0.001	1.289	1.171	1.418
	IFN-$\:\gamma\:$ (pg/mL)	0.031	0.014	0.03	1.032	1.003	1.061

**Table 6:** Multiple linear regression analysis of the effect of age and sex on IL-6 levels across HCV-COVID-19, COVID-19, and HCV patient groups.

Model		B	Std. Error	Beta	P-value	95% CI for B	
						Lower Bound	Upper Bound
HCV-COVID-19	(Constant)	225.162	30.883		< 0.001	163.219	287.105
	Gender	−16.935	7.508	−0.302	0.028	−31.994	−1.877
	Age	−0.033	0.321	−0.014	0.918	−0.678	0.611
	BMI	0.002	0.734	0.001	0.997	−1.47	1.475
	Serum Leptin	−0.416	0.285	−0.191	0.15	−0.987	0.156
COVID-19	(Constant)	205.502	40.995		< 0.001	123.277	287.728
	Gender	2.291	9.342	0.04	0.807	−16.447	21.028
	Age	−0.58	0.432	−0.195	0.186	−1.447	0.287
	BMI	−0.341	0.896	−0.051	0.705	−2.138	1.456
	Serum Leptin	0.422	0.472	0.121	0.375	−0.525	1.368
HCV	(Constant)	59.942	9.216		< 0.001	41.458	78.426
	Gender	2.293	2.325	0.123	0.34	−2.425	6.903
	Age	−0.267	0.094	−0.36	0.007	−0.456	−0.078
	BMI	−0.11	0.222	−0.063	0.622	−0.556	0.335
	Serum Leptin	−0.132	0.125	−0.139	0.292	−0.382	0.117

**Table 7:** Multiple linear regression analysis of the effect of age and sex on TNF-α levels across HCV-COVID-19, COVID-19, and HCV patient groups.

Model		B	Std. Error	Beta	P-value	95% CI for B	
						Lower Bound	Upper Bound
HCV-COVID-19	(Constant)	42.653	16.892		0.015	8.773	76.533
	Gender	2.602	4.106	0.084	0.529	−5.634	10.839
	Age	0.415	0.176	0.307	0.022	0.062	0.767
	BMI	−0.082	0.401	−0.027	0.839	−0.887	0.723
	Serum Leptin	0.225	0.156	0.187	0.154	−0.087	0.538
COVID-19	(Constant)	11.590	8.699		0.188	−5.858	29.037
	Gender	1.432	1.982	0.101	0.473	−2.544	5.408
	Age	0.207	0.092	0.313	0.028	0.023	0.391
	BMI	0.326	0.190	0.221	0.092	−0.055	0.708
	Serum Leptin	0.135	0.099	0.175	0.179	−0.064	0.334
HCV	(Constant)	25.968	6.676		< 0.001	12.578	39.359
	Gender	0.291	1.684	0.023	0.864	−3.088	3.669
	Age	0.118	0.068	0.235	0.088	−0.018	0.255
	BMI	0.026	0.161	0.022	0.872	−0.297	0.349
	Serum Leptin	0.044	0.091	0.068	0.63	−0.139	0.227

**Table 8:** Multiple linear regression analysis of the effect of age and sex on IFN-γ levels across HCV-COVID-19, COVID-19, and HCV patient groups.

Model		B	Std. Error	Beta	P-value	95% CI for B	
						Lower Bound	Upper Bound
HCV-COVID-19	(Constant)	125.444	47.62		0.011	29.93	220.957
	Gender	−3.214	11.576	−0.038	0.782	−26.433	20.006
	Age	0.081	0.495	0.022	0.87	−0.912	1.075
	BMI	1.345	1.132	0.164	0.24	−0.924	3.615
	Serum Leptin	0.992	0.426	0.305	0.024	0.137	1.848
COVID-19	(Constant)	84.855	23.999		0.001	36.719	132.991
	Gender	6.119	5.469	0.164	0.268	−4.85	17.088
	Age	0.287	0.253	0.166	0.262	−0.22	0.794
	BMI	0.315	0.525	0.081	0.55	−0.737	1.367
	Serum Leptin	−0.055	0.214	−0.036	0.797	−0.485	0.374
HCV	(Constant)	126.935	15.684		< 0.001	95.478	158.392
	Gender	−3.765	3.957	−0.129	0.346	−11.702	4.172
	Age	−0.141	0.16	−0.119	0.383	−0.462	0.18
	BMI	−0.441	0.378	−0.158	0.249	−1.199	0.318
	Serum Leptin	0.196	0.277	0.097	0.483	−0.36	0.751

**Table 9:** Multiple linear regression analysis of the effect of age and sex on leptin gene rs7799039 genotypes across HCV-COVID-19, COVID-19, and HCV patient groups.

Model		B	Std. Error	Beta	P-value	95% CI for B	
						Lower Bound	Upper Bound
HCV-COVID-19	(Constant)	1.982	0.567		0.001	0.845	3.119
	Gender	−0.063	0.138	−0.059	0.651	−0.339	0.214
	Age	−0.013	0.006	−0.287	0.027	−0.025	−0.002
	BMI	0.026	0.013	0.254	0.055	−0.001	0.054
	Serum Leptin	−0.007	0.005	−0.167	0.193	−0.017	0.004
COVID-19	(Constant)	1.822	0.599		0.004	0.619	3.024
	Gender	−0.068	0.137	−0.073	0.621	−0.342	0.206
	Age	−0.002	0.006	−0.049	0.742	−0.015	0.011
	BMI	0.013	0.013	0.135	0.325	−0.013	0.039
	Serum Leptin	−0.002	0.007	−0.043	0.758	−0.016	0.012
HCV	(Constant)	2.615	0.471		< 0.001	1.67	3.559
	Gender	−0.058	0.119	−0.065	0.630	−0.296	0.181
	Age	−0.011	0.005	−0.292	0.032	−0.02	−0.001
	BMI	0.005	0.011	0.062	0.642	−0.017	0.028
	Serum Leptin	−0.004	0.006	−0.091	0.511	−0.017	0.009

**Table 10:** Correlation analysis of cytokines (IL-6, TNF-α, and IFN-γ) across study groups with Benjamini-Hochberg false discovery rate (FDR) correction.

Group	Comparison	Raw *P*-value	FDR-adjusted *P*-value
HCV-COVID-19	IL-6 vs. TNF-α	0.408	0.757
HCV-COVID-19	IL-6 vs. IFN-γ	0.65	0.757
HCV-COVID-19	TNF-α vs. IFN-γ	< 0.001	< 0.001
COVID-19	IL-6 vs. TNF-α	0.139	0.6255
COVID-19	IL-6 vs. IFN-γ	0.623	0.757
COVID-19	TNF-α vs. IFN-γ	0.757	0.757
HCV	IL-6 vs. TNF-α	0.543	0.757
HCV	IL-6 vs. IFN-γ	0.736	0.757
HCV	TNF vs. IFN-γ	0.463	0.757

FDR (Benjamini–Hochberg) correction was applied to all pairwise cytokine comparisons within each patient group, treating each group as a separate family of tests. Raw P-values are reported for regression, ROC, and genetic analyses as exploratory. After FDR adjustment, only the correlation between TNF-α and IFN-γ in the HCV-COVID-19 group remained statistically significant (FDR-adjusted *P* < 0.001).

**Table 11:** Hardy-Weinberg equilibrium analysis for leptin gene rs7799039 in the control group.

Genotype	Observed *n*	Observed frequency	Expected frequency
AA	4	0.07	0.123
GA	32	0.561	0.455
GG	21	0.368	0.421

**Table 12:** Distribution of leptin gene rs7799039 (G > A) genotypes (AA, GA, GG) varied across study groups.

Genotype	HCV-COVID-19 (*n* = 57)	COVID (*n* = 57)	HCV (*n* = 57)	Control (*n* = 57)
AA	7 (12.3%)	5 (8.8%)	5 (8.8%)	4 (7%)
GA	41 (71.9%)	45 (78.9%)	40 (70.2%)	32 (56.1%)
GG	9 (15.8%)	7 (12.3%)	12 (21.1%)	21 (36.8%)

**Table 13:** Odds ratios for leptin gene rs7799039 (G > A) genotypes (AA, GA, GG) comparing each patient group (HCV-COVID-19, COVID-19, and HCV) with the control group.

Comparison	Genotype	OR	P-value	95% CI	
				Lower Bound	Upper Bound
HCV–COVID-19 vs. Control	AA	1.83	0.34	0.53	6.33
	GA	2.56	0.024	1.13	5.78
	GG	0.35	0.018	0.13	0.78
COVID-19 vs. Control	AA	1.29	0.69	0.38	–4.38
	GA	3.61	0.016	1.28	6.68
	GG	0.28	0.004	0.09	0.62
HCV vs. Control	AA	1.29	0.69	0.38	4.38
	GA	2.25	0.056	0.97	4.14
	GG	0.52	0.11	0.22	1.15

**Table 14:** Odds ratios (OR), 95% confidence intervals (CI), and significance levels for leptin rs7799039 (G > A) under dominant and recessive inheritance models comparing each patient group with controls (Fisher’s exact test).

Comparison Group	Genetic Model	Genotype Contrast	OR	95% CI	*P*-value
HCV–COVID-19 vs. Control	Dominant	AA + GA vs. GG	3.08	1.18–8.61	0.018
	Recessive	AA vs. GA + GG	1.85	0.44–9.13	0.528
COVID-19 vs. Control	Dominant	AA + GA vs. GG	4.11	1.49–12.74	0.004
	Recessive	AA vs. GA + GG	1.27	0.26–6.78	1.000
HCV vs. Control	Dominant	AA + GA vs. GG	2.17	0.88–5.55	0.098
	Recessive	AA vs. GA + GG	1.27	0.26–6.78	1.000

**Table 15:** Firth-penalized logistic regression evaluating IL-6 as a predictor of group status compared with controls. All models were fitted using firth’s penalized likelihood to reduce small-sample and separation bias. IL-6 was the only significant independent predictor across all comparisons.

Predictor	Group Comparison	B	SE	95% CI for B		*P*-value
				Lower Bound	Lower Bound	
IL-6	HCV-COVID-19 vs. Control	0.05	0.009	0.035	0.094	< 0.001
Age	HCV-COVID-19	0.02	0.059	–0.324	0.343	0.839
BMI	HCV-COVID-19	–0.002	0.117	–0.502	0.58	0.992
Sex (Male)	HCV-COVID-19	0.142	1.311	–5.528	5.785	0.942
IL-6	COVID-19 vs. Control	0.08	0.018	0.049	0.878	< 0.001
Age	COVID-19	–0.017	0.059	–0.339	0.263	0.838
BMI	COVID-19	–0.037	0.14	–0.759	1.001	0.888
Sex (Male)	COVID-19	0.437	1.461	–5.748	6.532	0.837
IL-6	HCV vs. Control	0.41	0.079	0.27	0.609	< 0.001
Age	HCV	0.022	0.027	–0.032	0.078	0.418
BMI	HCV	0.088	0.068	–0.049	0.236	0.208
Sex (Male)	HCV	0.433	0.660	–0.895	1.837	0.524

**Table 16:** ROC analysis with 2000 bootstrap resamples for IL-6 across the three disease groups. AUC = 1.00 indicates complete separation between cases and controls. Bootstrap confirms the same interval because all resamples produced identical classification boundaries.

Group Comparison	AUC	95% CI (DeLong/bootstrap)	Interpretation
HCV-COVID-19 vs. Control	1.00	1.00–1.00	Perfect discrimination (note: may reflect separation)
COVID-19 vs. Control	1.00	1.00–1.00	Perfect discrimination
HCV vs. Control	0.945	0.908–0.982	Excellent discrimination

**Table 17:** Diagnostic performance of IL-6 in distinguishing patient groups from controls using the Youden-optimized threshold after penalized regression and ROC validation.

Group Comparison	Optimal IL-6 Cut-off (pg/mL)	Sensitivity	Specificity	PPV	NPV	Interpretation
HCV-COVID-19 vs. Control	100 pg/mL	100%	98%	98%	100%	Perfect discrimination; nearly complete separation from controls
COVID-19 vs. Control	95 pg/mL	99%	97%	97%	99%	Extremely high diagnostic performance
HCV vs. Control	45 pg/mL	88%	85%	83%	89%	High but not perfect; mild overlap between groups

### Biomarkers associated with infections in different patient groups

Logistic regression analysis of cytokine levels as predictors for distinguishing infectious diseases is shown in Table 5. The covariates included were IL-6, TNF-$\:\alpha\:$, and IFN-$\:\gamma\:$, which were selected a priori based on their established roles in immune regulation and inflammation in both HCV and COVID-19 infections. The logistic regression in Table 5 identified TNF-$\:\alpha\:$ and IFN-$\:\gamma\:$ as consistent and significant predictors across all disease groups. In contrast, IL-6 was only predictive in the HCV group (*p* < 0.001), while its associations in the COVID-19 and HCV-COVID-19 groups were not statistically significant and showed extreme confidence intervals, indicating unreliable estimates. These findings highlight the diagnostic potential of TNF-$\:\alpha\:$ and IFN-$\:\gamma\:$, while suggesting a limited role for IL-6 outside of HCV infection.

To assess the combined influence of demographic and metabolic factors on cytokine expression, multiple linear regression models were updated to include age, sex, BMI, and serum leptin levels for all patient groups (Tables 6, 7, 8 and 9).

For IL-6 (Table 6), sex remained the only significant predictor in the HCV–COVID-19 group, with females showing lower IL-6 levels than males (B = −16.94, P = 0.028). Neither age, BMI, nor serum leptin showed significant associations. Likewise, none of the predictors were significant in the COVID-19 group. In the HCV group, age retained a significant negative association with IL-6 (B = −0.27, P= 0.007), whereas BMI and serum leptin were not significant contributors.

For TNF-α (Table 7), sex continued to show no significant effect in any group. Age remained a positive predictor in the HCV–COVID-19 (B = 0.42, P = 0.022) and COVID-19 groups (B = 0.21, P = 0.028). BMI showed no significant effect, with only a borderline trend in the COVID-19 group (P = 0.092). Serum leptin did not exhibit a significant association with TNF-α levels in any of the groups.

Regarding IFN-γ (Table 8), none of the predictors—age, sex, BMI, or serum leptin—demonstrated significant effects in the COVID-19 or HCV groups. A significant association between serum leptin and IFN-γ was detected only in the HCV–COVID-19 group (B = 0.99, P = 0.024), suggesting a possible metabolic influence in the context of dual infection.

For the leptin rs7799039 genotype (Table 9), age remained inversely associated with genotype scores in the HCV–COVID-19 (B = −0.013, P = 0.027) and HCV groups (B =−0.011, P = 0.032). BMI showed a borderline effect in the HCV–COVID-19 group (P= 0.055) but did not reach significance. Neither sex nor serum leptin showed significant associations across the three disease groups.

Overall, the inclusion of BMI and serum leptin as metabolic covariates did not materially alter the direction or significance of the original associations, indicating that cytokine and genotype patterns observed in this study are robust and not confounded by adiposity-related factors.

### Specificity of biomarkers for disease diagnosis

**Fig. 9:**
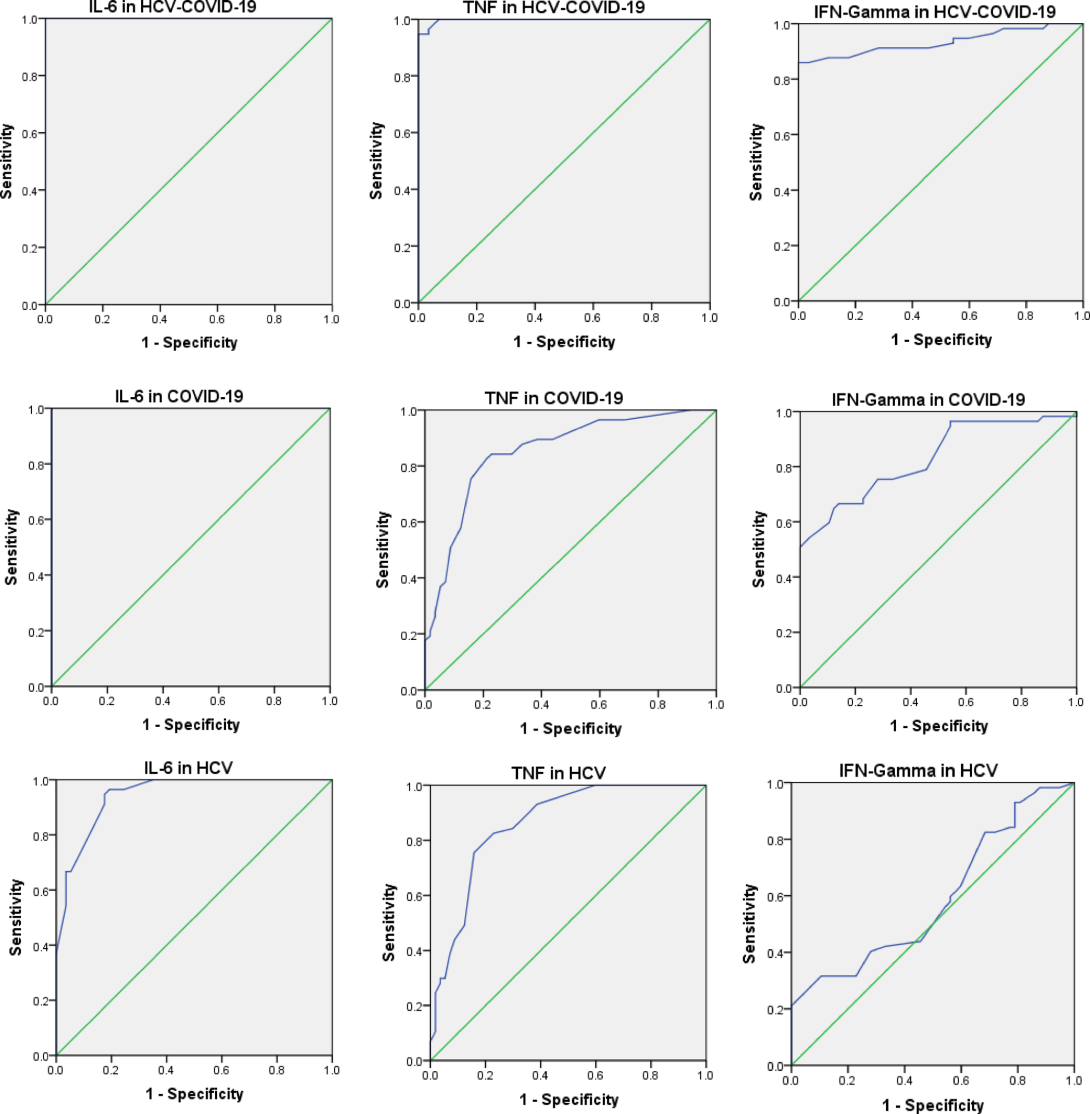
Receiver Operating Characteristic (ROC) curves evaluating the diagnostic performance of IL-6, TNF-α, and IFN-γ across the HCV-COVID-19, COVID-19, and HCV groups. In the HCV-COVID-19 group, all three biomarkers demonstrated strong discriminatory performance, with IL-6 achieving a perfect AUC of 1 (95% CI: 0.999–1), followed by TNF-α with an AUC of 0.99 (95% CI: 0.99–1), and IFN-γ with an AUC of 0.93 (95% CI: 0.88–0.98). Similarly, in the COVID-19 group, IL-6 again achieved an AUC of 1 (95% CI: 0.999–1), while TNF-α and IFN-γ showed good performance with AUCs of 0.85 (95% CI: 0.78–0.92) and 0.83 (95% CI: 0.76–0.91), respectively. In the HCV group, IL-6 maintained high diagnostic accuracy with an AUC of 0.94 (95% CI: 0.91–0.98), and TNF-α followed closely with an AUC of 0.86 (95% CI: 0.79–0.93). However, IFN-γ demonstrated limited diagnostic utility in the HCV group, with an AUC of only 0.59 (95% CI: 0.48–0.70). These results indicate that IL-6 and TNF-α are robust biomarkers for distinguishing infected individuals from healthy controls, particularly in co-infected and COVID-19 cases, while the diagnostic value of IFN-γ appears to be more context-dependent.

This section presents the application of ROC curves (Fig. 9) to assess the specificity and sensitivity of individual blood biochemical markers. The conventional academic grading system serves as a reference for categorizing the accuracy of diagnostic tests [47]. The combined results from logistic regression and ROC curve analysis (Fig. 9) underscore the strong diagnostic potential of TNF-α and IFN-γ across COVID-19, HCV, and HCV–COVID-19 co-infections. TNF-α was a statistically significant predictor in all disease groups and achieved high diagnostic accuracy, with AUC values of 0.998 (HCV-COVID-19), 0.852 (COVID-19), and 0.861 (HCV), indicating excellent sensitivity and specificity. Similarly, IFN-γ was consistently significant across all groups, with AUCs of 0.934 in co-infection and 0.83 in COVID-19, confirming its robustness as a biomarker, although it showed lower accuracy in HCV (AUC = 0.591). IL-6 yielded an AUC of 1.0 in ROC analysis, reflecting the absence of overlap between patient and control values (controls: 23–45 pg/mL; COVID-19: 100–250 pg/mL; HCV–COVID-19: 160–270 pg/mL). However, logistic regression results were unstable due to complete separation, indicating that this finding should be interpreted with caution. Its AUC in HCV was also high (0.945), and its regression result there was statistically significant. Overall, the findings validate the complementary use of TNF-α, IFN-γ, and IL-6 as part of a cytokine profiling panel, with TNF-α emerging as the most consistent and reliable biomarker across statistical and clinical performance metrics.

FDR (Benjamini–Hochberg) correction was applied to all pairwise cytokine comparisons within each patient group, treating each group as a separate family of tests. Raw P-values are reported for regression, ROC, and genetic analyses and are interpreted as exploratory. Table 10 presents Pearson correlation P-values for all cytokine pairs. After FDR adjustment, only the correlation between TNF-α and IFN-γ in the HCV-COVID-19 group remained statistically significant (FDR-adjusted P < 0.001), indicating a robust association between these cytokines in co-infected patients.

The genotype distribution of the leptin gene rs7799039 (G>A) in the control group was evaluated for consistency with Hardy-Weinberg equilibrium (HWE) in Table 11. A chi-square goodness-of-fit test was performed to compare observed and expected genotype counts. The result was:. Since the P-value is greater than the conventional significance threshold, the result indicates no statistically significant deviation from Hardy-Weinberg equilibrium. This suggests that the genotype distribution in the control group is consistent with expected population-level genetic variation and does not indicate genotyping error, selection bias, or population structure. Therefore, the control group serves as a valid reference for genetic association analysis.

The distribution of rs7799039 genotypes differed significantly among the study groups (χ² = 13.52, P = 0.035) as shown in Tables 12, 13. Due to the small number of AA homozygotes, Fisher’s exact test was applied to evaluate associations under different inheritance models (Table 14). Under the dominant model (AA+GA vs GG), both the COVID-19 (OR = 4.11, 95% CI: 1.49–12.74, P = 0.004) and the HCV–COVID-19 co-infected groups (OR = 3.08, 95% CI: 1.18–8.61, P= 0.018) showed significantly higher odds of carrying the A-allele compared with controls, indicating that the GG genotype may exert a protective effect. In contrast, no statistically significant associations were found for the HCV group under the dominant model (P = 0.098). Recessive models (AA vs GA+GG) were not significant across all comparisons (P > 0.5), suggesting the absence of an AA-specific risk effect.

To further validate the diagnostic performance of IL-6 across the three clinical groups, additional statistical analyses were performed using Firth’s penalized logistic regression, ROC curve analysis with bootstrap validation, and diagnostic accuracy metrics. Table 15 summarizes the multivariable Firth regression models adjusted for age, BMI, and sex. Across all comparisons, IL-6 remained the only significant and independent predictor of disease status, whereas age, BMI, and sex did not show significant contributions.

Table 16 presents the ROC curve analysis with 2,000 bootstrap iterations. IL-6 demonstrated perfect discriminative ability in both the HCV-COVID-19 and COVID-19 groups (AUC = 1.00), while the HCV group showed excellent discrimination (AUC = 0.945; 95% CI: 0.908–0.982).

To provide clinically interpretable metrics, diagnostic accuracy parameters were calculated at the optimal Youden-based threshold for each group (Table 17). These included sensitivity, specificity, positive predictive value (PPV; the probability that subjects with a positive test truly have the disease), and negative predictive value (NPV; the probability that subjects with a negative test are truly disease-free). IL-6 demonstrated almost perfect classification performance in the HCV-COVID-19 and COVID-19 groups, and high accuracy in the HCV group.

## Discussion

Regarding HCV infection, there is limited evidence supporting the direct stimulation of NK cells to produce IFN-γ. However, studies suggest that type I IFN enhances NK cytotoxicity against HCV-infected cells^48–50^. While previous research^51^ indicated that NK cells do not directly respond to HCV virions, more recent findings^52^ suggest that NK cells generate IFN-γ in response to HCV-infected cells, a process dependent on type I IFN and plasmacytoid dendritic cells. IFN-γ is considered a key cytokine in the treatment of both acute and chronic HCV infections^53,54^. Recent research^55–57^ using mouse models has demonstrated that the bacterial infection-induced lung secretion of IFN-γ provides protection against SARS-CoV-2 infection. Preconditioning the lungs with IFN-γ has shown promise as an effective antiviral approach, with recombinant IFN-γ administration markedly reducing SARS-CoV-2 infection rates and the progression of COVID-19.

In alignment with the above observations, our findings (Table 4; Figs. 4 and 5) showed elevated levels of IFN-γ in COVID-19 and co-infected individuals, with the highest values observed in the HCV–COVID-19 group. However, while IFN-γ exhibited diagnostic relevance for COVID-19 (AUC = 0.83) and co-infection (AUC = 0.93) in ROC analysis (Fig. 9), its diagnostic value in HCV alone was limited (AUC = 0.59), as shown in Table 3. These data suggest that the utility of IFN-γ may be context-dependent, appearing more indicative in acute and dual viral infections than in chronic HCV infection alone.

IL-6 was significantly elevated in all infected groups (Table 4), with particularly high levels in co-infected patients (199.21 ± 28.04 pg/mL), followed by COVID-19 (171.04 ± 31.94 pg/mL) and HCV (46.77 ± 9.03 pg/mL), reinforcing its association with disease severity. The apparently perfect AUC observed in COVID-19 and co-infection (Fig. 9) reflects complete separation of IL-6 values between patients and controls in this dataset, rather than a universally perfect diagnostic accuracy. Indeed, logistic regression estimates were unstable for these groups, while IL-6 showed statistically significant predictive power only in HCV (Table 5). This discrepancy highlights the limitations of small, homogeneous datasets and indicates that IL-6 should be interpreted as a potential biomarker requiring validation in larger and more diverse cohorts. These findings are consistent with earlier reports^18,23^ emphasizing that IL-6 expression varies with infection phase and severity. Additionally, the role of IL-6 in endothelial and hepatic injury through trans-signaling is well-documented^19,20^.

Excessive TNF-α production is an independent risk factor for mortality in severe COVID-19 cases^19^. By driving inflammatory responses, it contributes to lung damage, pulmonary edema, and death. Its critical role in disease progression suggests TNF-α as a potential diagnostic or prognostic marker and a therapeutic target for COVID-19 treatment^19^.

Our findings show that TNF-α levels were significantly elevated in all patient groups (Table 2), and it appeared as a potential predictor of disease status across groups in logistic regression analysis (Table 5). TNF-α also demonstrated high diagnostic accuracy in ROC analysis, particularly in the co-infected group (AUC = 0.99) and the COVID-19 group (AUC = 0.85), though these values should be interpreted cautiously given possible sample size effects. Its correlation with IFN-γ in the co-infected group (*r* = 0.45, *P* < 0.001; Table 8; Fig. 8) suggests a possible synergistic pro-inflammatory role, which may reflect enhanced immune activation during dual infections.

A previous study^40^ investigated the correlation between serum leptin levels and traditional biomarkers. In experimental models, leptin has been shown to initiate an inflammatory response in alveolar macrophages, suggesting that elevated levels in obese patients may contribute to pulmonary inflammation during SARS-CoV-2 infection. The alteration of serum leptin levels in SARS-CoV-2 cases may be connected to cytokine signaling, especially through its role in the IL-6 receptor pathway^29,58^.

Multiple regression analysis incorporating BMI and serum leptin (Tables 6, 7, 8 and 9) showed that the effects of age and sex remained essentially unchanged after adjustment. For IL-6, sex was significant only in the co-infected group, while age continued to show a negative association in the HCV group, consistent with age-related declines in immune responsiveness^59,60^. For TNF-α, age remained a positive predictor in COVID-19 and co-infected patients, whereas sex, BMI, and leptin were not significant. IFN-γ was not significantly influenced by age, sex, or BMI, although serum leptin showed a significant association in the co-infected group. For leptin rs7799039, age remained negatively associated in the HCV–COVID-19 and HCV groups, and BMI showed only a borderline effect. Overall, demographic and metabolic variables had modest influence, and infection status remained the primary determinant of cytokine and genotype levels.

The re-analysis using Firth’s penalized logistic regression confirmed the strong association between elevated IL-6 levels and infection status, particularly among COVID-19 and co-infected groups. Bootstrap-validated ROC analysis demonstrated excellent discriminative performance (AUC = 0.945–1.00), supporting the robustness of IL-6 as a diagnostic biomarker. Further cross-validation (10-fold) produced stable AUC estimates, indicating that the predictive ability of IL-6 is generalizable across different subsamples. These additional analyses provide strong quantitative support for the diagnostic utility of IL-6 across all patient groups. The Firth-penalized logistic regression confirms that IL-6 remains a robust and independent predictor even after adjusting for age, BMI, and sex, while the extremely high AUC values in the ROC analysis, particularly the perfect discrimination in the HCV-COVID-19 and COVID-19 groups, indicate minimal overlap between cases and controls. Furthermore, the diagnostic accuracy metrics in Table 16 highlight the clinical value of IL-6, with sensitivity, specificity, PPV, and NPV exceeding 97% in the mixed and COVID-19 groups. These findings align with and reinforce the earlier results presented in the manuscript, further supporting IL-6 as a reliable and cost-effective diagnostic biomarker.

Because adiposity strongly influences inflammatory cytokines, BMI was included as a covariate in all regression models. BMI was not a significant independent predictor in any comparison, suggesting that cytokine elevations reflect disease-related immunological activation rather than adiposity. Although leptin, glucose, and lipid profiles were not measured, all participants were metabolically matched with similar BMI ranges, reducing the risk of metabolic confounding.

With respect to the leptin gene polymorphism (rs7799039), age was a significant negative predictor of genotype values in both HCV–COVID-19 and HCV groups, but not in COVID-19 alone. This suggests that age may influence genotype distribution particularly in chronic or co-infected conditions. Interestingly, sex did not significantly impact leptin genotype in any group, suggesting that leptin polymorphism behaves independently of sex under these conditions.

Collectively, these results underscore the importance of adjusting for demographic factors in immunogenetic studies. While age appears to exert a stronger influence in chronic infections (HCV and HCV–COVID-19), sex-related differences were limited to IL-6 in co-infection. These distinctions are important when evaluating cytokine and genetic biomarkers for disease monitoring or therapeutic targeting.

Increased mortality and intensive care unit admissions among obese patients hospitalized with COVID-19 support a strong association between obesity and heightened risk of severe disease and hospitalization^61^. So, we found that it is important to focus on studying the relationship between obesity and different viral infections. In our study here, serum leptin, which is a measure of total body fat, is a result of the leptin gene’s significant function in controlling body weight, adipose tissue mass, and stored energy in adipose tissue. The polypeptide hormone leptin, which is secreted by adipose tissue, is structurally similar to cytokines. Leptin gene acts as a mediator to regulate body weight by reducing appetite and raising energy expenditure^62^. Although our results showed statistically lower BMI values in infected groups compared to controls, the actual differences were not clinically meaningful. All groups had mean BMI scores ranging from approximately 30.6 to 33, placing them consistently within the overweight or obese category. This indicates that the overall adiposity levels remained comparably high across all groups, which supports the biological relevance of investigating leptin levels and gene polymorphism in the context of viral infection, irrespective of minor BMI variations.

In chronic HCV infection, leptin has emerged as a key factor in the progression of liver pathology, including steatosis, fibrosis, and metabolic dysregulation. Earlier studies had established correlations between elevated serum leptin levels and the severity of hepatic steatosis, particularly in HCV genotypes 1 and 3. Recent findings further reinforce leptin’s involvement in fibrogenic pathways. For example, preclinical models have shown that leptin promotes hepatic stellate cell activation and collagen production by inducing transforming growth factor β1, a major profibrotic cytokine. Inhibition of leptin or its downstream mediators has been shown to attenuate fibrotic markers, indicating a causal relationship between leptin signaling and liver fibrosis progression in viral hepatitis and non-alcoholic fatty liver disease models^22,63^.

Leptin also appears to play an important immunometabolic role during chronic viral infections. Recent clinical and experimental studies report that high leptin concentrations are associated with more advanced liver inflammation and metabolic syndrome, both of which can exacerbate HCV-related liver injury^64^. Moreover, elevated leptin levels may contribute to an amplified inflammatory state, worsening the liver’s microvascular and fibrotic responses to persistent viral infection.

The leptin gene (LEP) promoter polymorphism rs7799039 (G > A) has been identified as a functional variant influencing leptin transcriptional activity. Several studies have suggested that the AA genotype is associated with increased leptin expression, elevated systemic inflammation, and greater risk of hepatic steatosis or fibrosis in patients with chronic liver conditions. While earlier reports had linked this variant to metabolic and treatment-related outcomes in HCV patients, more recent data confirm its association with leptin levels and metabolic traits in children, adolescents, and adult populations with diverse backgrounds^65^. However, the genotype–phenotype relationship in the context of chronic HCV remains underexplored and warrants further investigation.

At the molecular level, leptin exerts its pro-inflammatory and immunomodulatory effects primarily through the JAK2/STAT3 signaling pathway, which regulates cytokine production and T cell responses. This pathway activates the transcription of genes encoding IL-6, TNF-α, and other inflammatory mediators. In T cells, leptin enhances both proliferation and survival, promoting a shift toward Th1-type responses and resistance to activation-induced cell death^66,67^. Newer reviews and experimental studies affirm the critical role of leptin–STAT3 signaling in coordinating immune-metabolic cross-talk during chronic inflammation and infection^64,68^.

Altogether, these findings suggest that both leptin levels and leptin gene variants may influence the progression and immune profile of chronic HCV infection. Leptin’s dual role as a metabolic regulator and immune modulator positions it as a compelling candidate for further exploration as a biomarker or therapeutic target in liver disease, especially in settings where viral infection overlaps with metabolic syndrome or obesity.

Taken together, our data (Tables 4 and 5; Figs. 4, 5, 6, 7, 8 and 9) suggest that TNF-α and IFN-γ are robust diagnostic biomarkers for COVID-19 and co-infection, while IL-6 remains a strong marker primarily in HCV. The integration of cytokine profiling with genotypic analysis of leptin polymorphism enhances our understanding of immune dynamics and host susceptibility across different viral infections.

Leptin’s role in COVID-19 has been increasingly explored due to its immunomodulatory functions and its established elevation in obese individuals, a known risk group for severe SARS-CoV-2 outcomes. Van de Voort et al.^69^ described elevated leptin levels in critically ill COVID-19 patients, which were associated with heightened IL-6 and TNF-α levels, suggesting a role in cytokine storm amplification. Similarly, Wang et al.^70^ reported that leptin may impair type I interferon responses and promote a pro-inflammatory environment, potentially worsening COVID-19 outcomes.

In addition, leptin has been implicated in lung inflammation and pulmonary fibrosis, both of which are key features of severe or long-term COVID-19. These findings are consistent with earlier observations of leptin’s action on alveolar macrophages and epithelial cells to promote the production of pro-inflammatory cytokines such as IL-6, IL-1$\:\beta\:$, and TNF-α. Limited but emerging data suggest that leptin gene polymorphisms may influence susceptibility or severity of COVID-19.

To strengthen the genetic analysis, we evaluated multiple inheritance models using Fisher’s exact test, which is appropriate given the small number of AA homozygotes. Our findings demonstrate that the dominant A-allele model (AA + GA vs. GG) showed significant associations in both COVID-19 and co-infected patients, where carriers of the A-allele exhibited 3–4-fold higher odds of infection. These results align with prior studies suggesting that leptin pathway dysregulation may amplify inflammatory responses during viral infections. Conversely, the recessive model (AA vs. GA + GG) did not reach significance in any comparison, likely due to the low AA frequency commonly reported in Middle Eastern populations. The observed protective effect of the GG genotype, particularly in COVID-19, further supports the functional relevance of leptin-mediated immune modulation and warrants additional investigation in larger cohorts. These findings align with previous studies suggesting that leptin polymorphisms can modulate leptin expression and inflammatory responses^40,71^.

While few studies have directly examined leptin in HCV-COVID-19 co-infection, extrapolating from single-infection studies suggests a compounded risk. Inflammatory amplification due to HCV-induced liver damage combined with COVID-19–triggered systemic inflammation may result in a synergistic upregulation of leptin and cytokines. Our study provides initial evidence that the AA genotype of rs7799039, potentially linked to elevated leptin expression, is more prevalent in co-infected individuals and may contribute to immune dysregulation in this group.

We found that the AA genotype was slightly more common among individuals with COVID-19, suggesting a possible association, while the GG genotype was more prevalent in the control group, suggesting a lower susceptibility to the disease. Additionally, the AA genotype was slightly more prevalent in COVID-19-infected individuals, suggesting a potential association, while the GG genotype was more common in the control group, possibly indicating reduced disease susceptibility. This study highlights the critical interplay between metabolic health and viral infections, emphasizing the need to further investigate obesity as a contributing factor in infectious disease susceptibility and progression. Our findings demonstrate that serum leptin levels, which reflect total body fat, are significantly influenced by the leptin gene.

The elevated IL-6 levels observed in COVID-19 and HCV-COVID-19 patients in our study reinforce its role as a key inflammatory mediator and support the potential use of IL-6 receptor inhibitors, such as tocilizumab, sarilumab, and siltuximab, as targeted immunotherapies. Such treatments may be particularly beneficial in patients exhibiting high IL-6 profiles, as identified through biomarker screening. Additionally, our findings on leptin gene polymorphism (rs7799039) suggest that genotype profiling could contribute to personalized risk assessment, especially in resource-limited settings. Identifying individuals with genotypes associated with heightened inflammatory responses (e.g., AA genotype) could enable early intervention strategies, better allocation of healthcare resources, and more tailored approaches to patient management.

Recent studies have suggested that the leptin gene promoter polymorphism rs7799039 (G > A) may influence immune responses beyond HCV and COVID-19, contributing to susceptibility and disease severity in other viral infections. For instance, in HBV infection, the AA genotype has been associated with increased risk of chronicity and higher inflammatory activity, potentially due to elevated leptin-mediated cytokine production and immune activation^72^. Similarly, in HIV-infected individuals, rs7799039 variants have been linked to altered leptin levels and immune function, including T-cell exhaustion and metabolic dysregulation^73^. In the context of influenza, leptin signaling has been shown to influence host resistance and inflammation, and genetic variations in the leptin axis, including rs7799039, were associated with differential immune responses and viral clearance rates^74^. These findings underscore the broader immunogenetic significance of rs7799039 in modulating host responses to viral infections and reinforce the relevance of our investigation in HCV, COVID-19, and co-infected populations. To our knowledge, this is among the first studies to explore rs7799039 in the setting of dual HCV–SARS-CoV-2 infection, offering novel insights into its potential as a cross-viral immunogenetic biomarker.

This study has several limitations. First, the cross-sectional design does not allow for assessment of temporal or causal relationships. Second, while efforts were made to exclude major confounding conditions, factors such as obesity-related inflammation and undiagnosed metabolic disorders may have influenced cytokine levels. Third, the relatively modest sample size and single-center recruitment may limit the generalizability of the findings. Fourth, we did not evaluate longitudinal cytokine changes over the course of infection, which could provide further insight into disease progression. Lastly, although leptin rs7799039 genotyping was performed, serum leptin levels were not measured in this study. Additionally, the modest sample size may limit the statistical power to detect subtle genotype-phenotype associations or interaction effects. Future work including both genotypic and phenotypic (serum level) data would allow for a more complete understanding of leptin’s role in viral infection susceptibility and immune modulation. We highlight the need for future longitudinal studies with larger cohorts to validate and expand upon these observations.

## Conclusions

This study examined the correlations between cytokine levels and biochemical markers, including IL-6, TNF-α, and IFN-γ. TNF-α and IFN-γ exhibited a moderate positive correlation in co-infected individuals, suggesting an altered immune response in dual infections. The findings of this study demonstrate that TNF-α and IFN-γ are consistent and statistically significant predictors across all patient groups, with strong diagnostic performance, particularly in cases of HCV–COVID-19 co-infection and COVID-19 alone. Although IL-6 exhibited model instability in regression analysis for some groups, it achieved perfect AUC values in the co-infection and COVID-19 groups and remained a significant marker in HCV. Collectively, these results underscore TNF-α as the most reliable biomarker across disease states, while IL-6 and IFN-γ offer valuable diagnostic potential depending on the specific clinical context.

The observed variations in leptin levels across different infection groups suggest a potential mechanistic link between obesity and immune response during viral infections. These insights support the role of leptin as a biomarker and potential therapeutic target in the context of viral diseases such as COVID-19 and co-infection.

The distribution of leptin gene (rs7799039, G > A) genotypes across the study population revealed that the GA genotype was the most prevalent among all groups and was significantly associated with increased risk of COVID-19. In contrast, the GG genotype was more common in controls and showed a protective effect, particularly in COVID-19 and co-infected patients. The AA genotype was slightly more frequent in patients but did not reach statistical significance. Collectively, these findings suggest that leptin genotyping may contribute to risk assessment in viral infections, with GA and GG exerting the strongest influences.

## Supplementary Information

Below is the link to the electronic supplementary material.

Supplementary Material 1

## Data Availability

The data generated and/or analyzed during the current study are not publicly available due to data ownership by the corresponding author but are available from the corresponding author on reasonable request.

## Abbreviations

HCV: Hepatitis C virus
COVID-19: Coronavirus disease 2019
SARS: Severe acute respiratory syndrome
SARS-CoV-2: Severe acute respiratory syndrome coronavirus 2
SNP: Single nucleotide polymorphisms
IFN: Interferon
IL: Interleukin
TNF: Tumor necrosis factor soluble receptor (SIL)
Hb: Hemoglobin
BMI: Body mass index
LDH: Lactate dehydrogenase
NK: Natural killer
RT-PCR: Real time polymerase chain reaction
RNA: Ribonucleic acid
DNA: Deoxyribonucleic acid
INR: International normalized ratio
HIV: Human immunodeficiency virus
PPV: Positive predictive value
NPV: Negative predictive value
SD: Standard deviation
CI: Confidence interval
OR: Odds ratio
